# Transcriptomic analysis of intestinal organoids, derived from pigs divergent in feed efficiency, and their response to *Escherichia coli*

**DOI:** 10.1186/s12864-024-10064-0

**Published:** 2024-02-13

**Authors:** Ole Madsen, Roxann S.C. Rikkers, Jerry M. Wells, Rob Bergsma, Soumya K. Kar, Nico Taverne, Anja J. Taverne-Thiele, Esther D. Ellen, Henri Woelders

**Affiliations:** 1 Animal Breeding & Genomics, Wageningen University & Research, PO Box 338, Wageningen, 6700 AH the Netherlands; 2 Topigs Norsvin, Schoenaker 6, 6641 SZ Beuningen, the Netherlands; 3 Animal Nutrition, Wageningen University & Research, PO Box 338, Wageningen, 6700 AH the Netherlands; 4 Host-Microbe Interactomics, Wageningen University & Research, PO Box 338, Wageningen, 6700 AH the Netherlands

**Keywords:** Feed efficiency, Gene expression, Immunity, Intestinal organoids, Pigs

## Abstract

**Background:**

There is increasing interest in using intestinal organoids to study complex traits like feed efficiency (FE) and host-microbe interactions. The aim of this study was to investigate differences in the molecular phenotype of organoids derived from pigs divergent for FE as well as their responses to challenge with adherent and invasive *Escherichia coli* (*E. coli*).

**Results:**

Colon and ileum tissue from low and high FE pigs was used to generate 3D organoids and two dimensional (2D) monolayers of organoid cells for *E. coli* challenge. Genome-wide gene expression was used to investigate molecular differences between pigs that were phenotypically divergent for FE and to study the difference in gene expression after challenge with *E. coli*. We showed, (1) minor differences in gene expression of colon organoids from pigs with low and high FE phenotypes, (2) that an *E. coli* challenge results in a strong innate immune gene response in both colon and ileum organoids, (3) that the immune response seems to be less pronounced in the colon organoids of high FE pigs and (4) a slightly stronger immune response was observed in ileum than in colon organoids.

**Conclusions:**

These findings demonstrate the potential for using organoids to gain insights into complex biological mechanisms such as FE.

**Supplementary Information:**

The online version contains supplementary material available at 10.1186/s12864-024-10064-0.

## Background

Adult stem cell-derived organoids hold great promise as in vitro models to study animal biology, including farm animal species [1]. Intestinal organoids are self-renewing and self-organizing three dimensional (3D) multicellular structures and contain, similar cell-types, structure and functionality as the organ or tissue they are derived from [2–4]. Moreover, organoids generated from adult stem cells retain their location specific patterns of expression [4, 5]. This makes it possible to study intestinal functionality in a reductionist way and to control exposure to nutrients, microorganisms, metabolites or effectors of the innate immune system. Thus, organoids are a good intermediate between high throughput/low complexity monolayer cell cultures and low throughput/high complexity animal models [6]. The 3D geometrical structure of the organoids grown in Matrigel form 3D structures meaning direct access to the apical surface of the epithelium requires injection. Therefore, 2D monolayer models have been developed for human and porcine 3D organoids, enabling access to the apical mucosal side as well as the basal serosal surface of the epithelium [3, 7, 8].

Comparison of the gene expression profiles of organoids in different conditions, or derived from different animals with distinct phenotypes, could reveal differences at a molecular level related to the condition or trait of interest. Thus, organoids can be used to investigate the potential contribution of molecular or functional tissue phenotypes to complex animal traits measured in vivo*.* An example of an important complex trait in the pig industry is feed efficiency (FE). Improvement of FE could reduce the feed costs and simultaneously improve the sustainability of the pig industry. Many factors affect this complex trait, which are excellently covered elsewhere, e.g. in the book “Feed Efficiency in Swine” [9]. It has been shown that an immune response to an infectious or noninfectious challenge can reduce FE [10–13]. The metabolic changes that occur as a result of inflammation have significant physiological costs [10, 13]. In addition, mucosal inflammation can seriously compromise intestinal functionality and nutrient absorption. Even low-grade intestinal inflammation may affect the health and total surface area of intestinal villi [14–17] and can increase the passage rate of digesta along the gastro-intestinal tract, reducing the time available for nutrients to be digested and absorbed [18].

There is evidence that animals with high FE are effective at maintaining immune homeostasis, thereby minimizing metabolic cost and reduced intestinal functionality. For instance, a number of studies [19–22] found that high FE pigs had a lower expression of immune related genes and lower rectal temperature than low FE pigs, after a lipopolysaccharide (LPS) challenge. High FE pigs also had lower feed intake, higher fecal pH, less acetate in colonic digesta, and higher populations of Lactobacillus spp. in the cecum [19, 23] than low FE efficiency pigs, suggesting a slower passage of digesta. In addition, high FE pigs had higher expression of genes for digestive enzymes and nutrient transporters and higher feed digestibility, shorter crypts, and greater mucosal permeability [23, 24].

Some aspects of gut functionality, for instance transepithelial nutrient transport and the presence of transporters, have been previously studied in intestinal organoids [4]. Stem cell derived intestinal organoids can be used to study innate immune responses to pathogens or to pathogen associated molecular patterns (PAMPs), even though they do not contain cells of the immune system. Higher expression of chemokines and inflammatory cytokines in low FE pig organoids would provide further evidence to support the hypothesis that inflammatory responses differ between high and low FE pigs and affect the FE phenotype.

In this study, we measured genome-wide gene expression responses of 2D organoid monolayers from high and low FE pigs challenged with adherent and invasive *Escherichia coli* (*E. coli*) pathobionts. The objective of this study was to investigate 1) to what extent colon organoids derived from low and high FE pigs differ in gene expression profiles, 2) how colon and ileum organoids respond to an *E. coli* challenge, and 3) whether colon organoids derived from low and high FE pigs differ in their response.

## Results

### Descriptive statistics of colon organoids

**Fig. 1:**
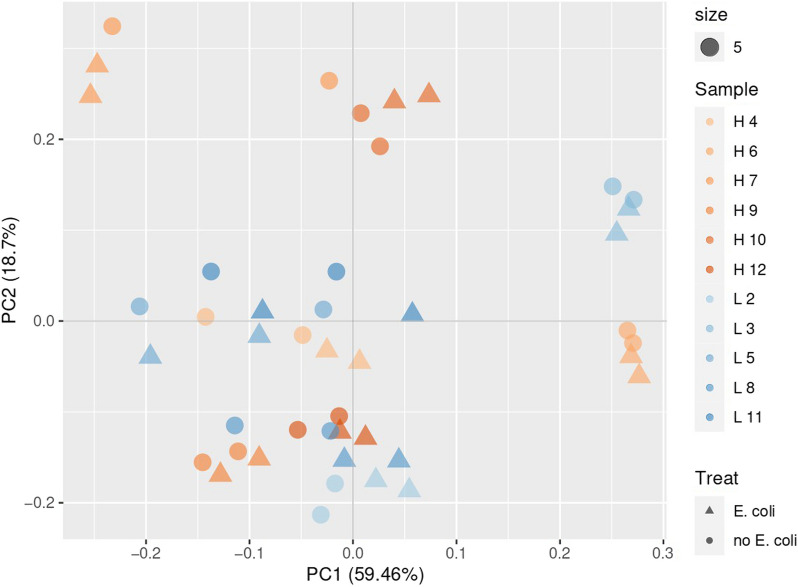
Principal component analysis plot of the colon organoid samples based on RSEM transcript per million (TPM) estimates (blue like colors = low FE (L), orange like colors = high FE (H), circles = unchallenged organoids and triangles = *E. coli* challenged organoids)

Whole genome RNA sequencing of 44 colon organoid samples (6 × 2 high FE unchallenged, 6 × 2 high FE *E. coli* challenged, 5 × 2 low FE unchallenged and 5 × 2 low FE *E. coli* challenged), were analyzed to determine and quantify gene expression profiles. RNA reads were aligned to the pig reference genome using STAR. For all 44 samples 31,752,427 ± 2,156,623 (mean + SD) uniquely mapped reads were identified, which was 95.30% ± 0.54% (mean ± standard deviation (SD)) of the total number of reads after trimming (Additional file 1 for detailed alignment results of the colon organoids). Clustering analysis based on global gene expression did not reveal any specific clustering related to FE or challenged versus unchallenged samples (Fig. 1), suggesting that the global gene expression is not related to the FE phenotype or to an *E. coli* challenge. Furthermore, the biological replicates of individual animals clustered together in general, regardless of their FE phenotype or *E. coli* challenge, indicating a general lower between-replicate variation than between-animal variation. One of the samples of animal 7 (high FE) was noticeably different compared to the samples of the other animals.

### Colon specific genes

104 genes were retrieved from the TiGER database as being colon specific. Of these, 79 genes were expressed (TPM > 1) in the unchallenged colon organoids (Additional file 2; Table 1 for subset of genes) suggesting that the colon organoids have many of the similar cell types as the tissue they were derived from. In general, the expression of these colon specific genes is similar between high and low FE derived organoids suggesting homogeneous cell composition among high and low FE derived organoids.

**Table 1:** Expression (TPM) in colon organoids of a subset of genes that are expected to be expressed in the porcine colon and/or that had been studied in a study on ex vivo LPS challenge of porcine colon tissue [20, 22]. A number of these genes were not (or hardly) expressed in the colon organoids are in bold (TPM values < 1). Genes that showed a significant response to E coli challenge are shown underlined and bold. Full list of expression of colon specific genes retrieved from TiGER database can be found in Additional file 2

	Genes	High FE	Low FE	High FE challenged	Low FE challenged
Expected colon genes	*ACTA2*	**0.08 ± 0.08**	**0.11 ± 0.12**	**0.08 ± 0.12**	**0.07 ± 0.10**
	*ANTXR1*	1.62 ± 1.12	2.49 ± 1.93	2.11 ± 1.28	3.34 ± 2.66
	*CALD1*	54.72 ± 36.22	59.76 ± 29.02	67.33 ± 38.99	66.70 ± 33.09
	*CDX2*	17.33 ± 11.11	14.66 ± 10.12	11.68 ± 10.28	9.14 ± 7.05
	*CFTR*	7.10 ± 4.71	6.92 ± 4.52	10.96 ± 5.95	9.58 ± 6.07
	*CHGA*	2.67 ± 3.79	2.79 ± 3.26	2.26 ± 3.79	2.93 ± 4.35
	*GLP2R*	**0.70 ± 0.35**	**0.82 ± 0.30**	**0.51 ± 0.25**	**0.52 ± 0.20**
	*HOXA3*	**0.54 ± 0.38**	**0.69 ± 0.30**	**0.65 ± 0.46**	**0.64 ± 0.26**
	*HOXD9*	1.09 ± 0.59	1.06 ± 0.73	**0.97 ± 0.47**	**0.62 ± 0.25**
	*KRT20*	34.19 ± 30.78	35.89 ± 28.65	22.61 ± 23.80	27.23 ± 19.80
	*MTOR*	13.14 ± 2.18	14.02 ± 2.66	16.99 ± 3.50	16.97 ± 2.33
	*NIFK*	104.67 ± 12.52	106.96 ± 10.46	96.14 ± 13.19	96.23 ± 10.89
	*PECAM1*	1.33 ± 0.81	1.18 ± 0.20	1.17 ± 0.68	**0.97 ± 0.18**
	*SDC1*	232.00 ± 44.53	240.94 ± 41.92	263.98 ± 54.44	240.54 ± 56.73
	*SLC16A1*	115.71 ± 24.37	118.54 ± 18.55	131.42 ± 35.62	129.58 ± 18.90
	*SLC44A4*	152.29 ± 73.96	124.00 ± 53.94	153.80 ± 88.09	124.18 ± 55.10
	*SLC5A1*	9.24 ± 2.91	6.61 ± 1.85	8.96 ± 2.74	7.36 ± 2.43
	*SLC9A3*	1.11 ± 1.20	**0.41 ± 0.34**	**0.91 ± 1.61**	**0.25 ± 0.15**
	*WNT5B*	3.89 ± 2.53	4.81 ± 3.25	4.62 ± 2.47	4.94 ± 3.67
Immunity related genes	*FOS (AP1)*^*a*^	32.09 ± 5,86	39.63 ± 9,08	92,81 ± 28,12	97,19 ± 34,11
	*FOSB (AP1)*^*a*^	**0,14 ± 0,19**	**0,13 ± 0,11**	**0,6 ± 0,56**	**0,59 ± 0,25**
	*FOSL1 (AP1)*^*a*^	42,19 ± 22,80	48,76 ± 20,08	78,84 ± 38,01	88,87 ± 20,62
	*FOSL2 (AP1)*^*a*^	57,93 ± 10,55	57,41 ± 7,73	74,53 ± 8,87	73,94 ± 5,42
	*JUNB (AP1)*^*a*^	176,52 ± 25,24	193,03 ± 43,28	315,41 ± 156,88	306,77 ± 91,08
	*CLDN2*	**0.94 ± 0.51**	1.02 ± 0.73	**0.55 ± 0.36**	**0.72 ± 0.44**
	***CXCL8***	183.83 ± 123.61	221.65 ± 198.37	1968.36 ± 599.07	2549.55 ± 724.44
	*FFAR2*	**0**	**0**	**0**	**0**
	*IFNG*	**0**	**0**	**0**	**0**
	***IL1A***	16.97 ± 8.84	15.44 ± 2.61	110.22 ± 49.75	112.59 ± 46.72
	*IL6*	**0.34 ± 0.28**	**0.72 ± 0.50**	**0.63 ± 0.69**	1.05 ± 0.96
	*IL10*	**0**	**0**	**0**	**0**
	*JAK2*	23.10 ± 6.71	20.83 ± 1.89	21.23 ± 3.86	19.02 ± 1.70
	*NFAM1*	**0**	**0**	**0.01 ± 0.03**	**0**
	*SOCS1*	2.31 ± 0.99	1.48 ± 0.51	2.25 ± 1.10	2.14 ± 1.33
	*SOCS3*	3.77 ± 1.34	3.78 ± 0.56	3.39 ± 1.95	2.83 ± 0.84
	*SOCS4*	13.79 ± 1.94	13.92 ± 1.16	12.22 ± 1.59	11.76 ± 0.98
	*SOCS5*	7.29 ± 0.64	7.29 ± 0.72	7.48 ± 1.12	6.81 ± 0.79
	*SOCS6*	30.75 ± 6.90	31.16 ± 4.35	28.65 ± 4.65	28.33 ± 2.15
	*TLR1*	2.10 ± 0.96	2.02 ± 0.82	1.73 ± 0.67	1.65 ± 0.71
	***TLR4***	9.73 ± 1.95	9.92 ± 2.26	15.32 ± 4.78	15.04 ± 3.81
	*TLR6*	1.28 ± 0.49	**0.91 ± 0.46**	**0.95 ± 0.45**	**0.72 ± 0.24**
	*TLR8*	**0.00 ± 0.01**	**0.00 ± 0.01**	**0.01 ± 0.01**	**0.01 ± 0.01**
	***TNF***	**0.63 ± 0.52**	**0.57 ± 0.49**	20.64 ± 6.99	24.89 ± 20.35
	*TRAM1*	163.73 ± 24.60	165.57 ± 23.23	143.78 ± 15.86	151.46 ± 12.43

^a^The gene indicated as AP1 in Vigors et al. (2019) is not available as such in the current pig genome annotation. Based on the description in Vigors et al. (2019) as “transcription factor AP1/JUN” we included the five subunit genes for *AP1* (*FOS*, *FOSB*, *FOSL1*, *FOSL2*, *JUNB*) as part of AP1 complex

Expression of genes previously found to be related with FE and/or immunity after an immune challenge [20, 22] are shown in Table 1. Most of these genes are expressed, as only 5–6 genes were not above the expression threshold, and variation in gene expression levels between the groups reveals little variation between high and low FE. Thus, based on genes expressed in the derived colon organoids they appear to be a suitable model to examine genes in relation to FE and/or innate immune response.

### Difference between unchallenged low and high FE colon organoids

**Fig. 2:**
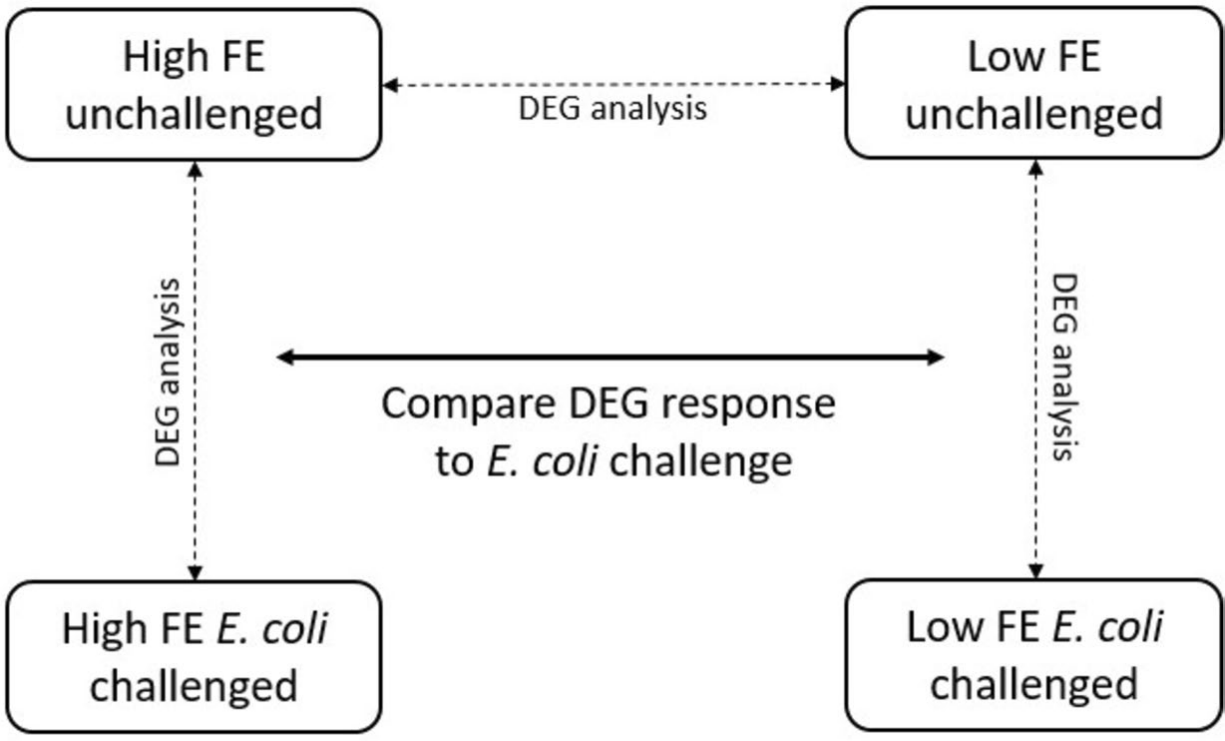
Schematic visualization of the experimental set up and gene expression comparisons. Differential expression was analyzed for high versus low FE unchallenged organoids. For both low and high FE groups, comparison of challenged versus unchallenged organoids was performed. Lastly, the response to the challenge in the two groups were compared to find similarities and differences in the up or down regulation of genes in the two groups

**Fig. 3:**
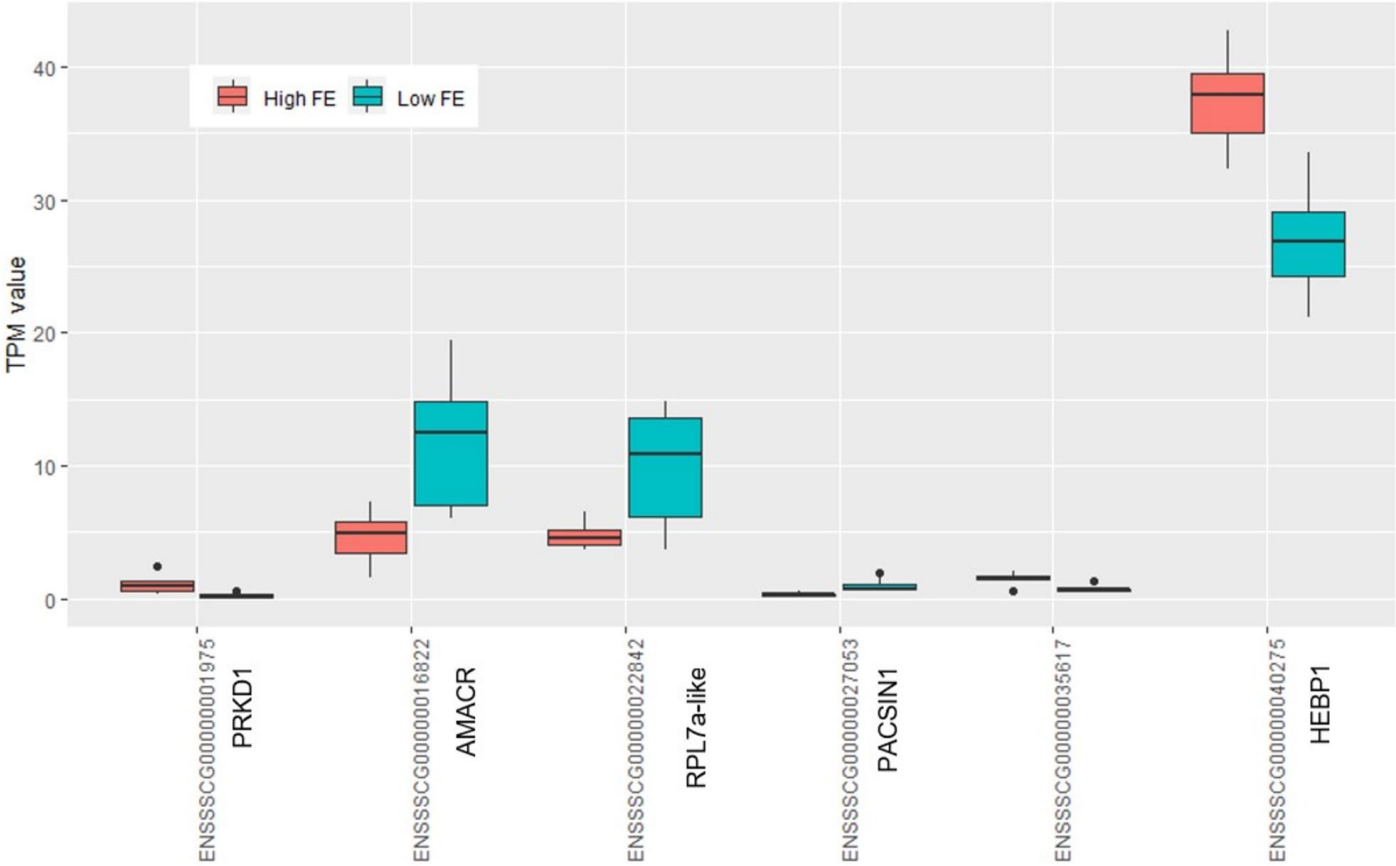
TPM values of the six differentially expressed genes between unchallenged low and high FE colon organoids. *ENSSSCG00000035617* is an uncharacterized novel pig gene

DESeq and EdgeR were used to determine DEGs between the four groups (Fig. 2). Only genes differentially expressed by both programs were considered differential expressed and used in functional gene enrichment analysis. From a total of 14,435 expressed genes only six genes were found to be significantly differentially expressed between low and high FE colon organoids (Fig. 3 and Additional file 3). *PRKD1*, *ENSSSCG00000035617*, and *HEBP1* were expressed higher, while *PACSIN1*, *AMACR*, and *RPL7a-like* (*ENSSSCG00000022842*) were expressed lower in the High FE group than in the Low FE group. Functional enrichments of these six genes did not result in any GO enrichment suggesting that at the gene expression level there is limit difference between the low and high FE phenotypes.

### Gene expression in colon organoids challenged with *E. coli*

**Fig. 4:**
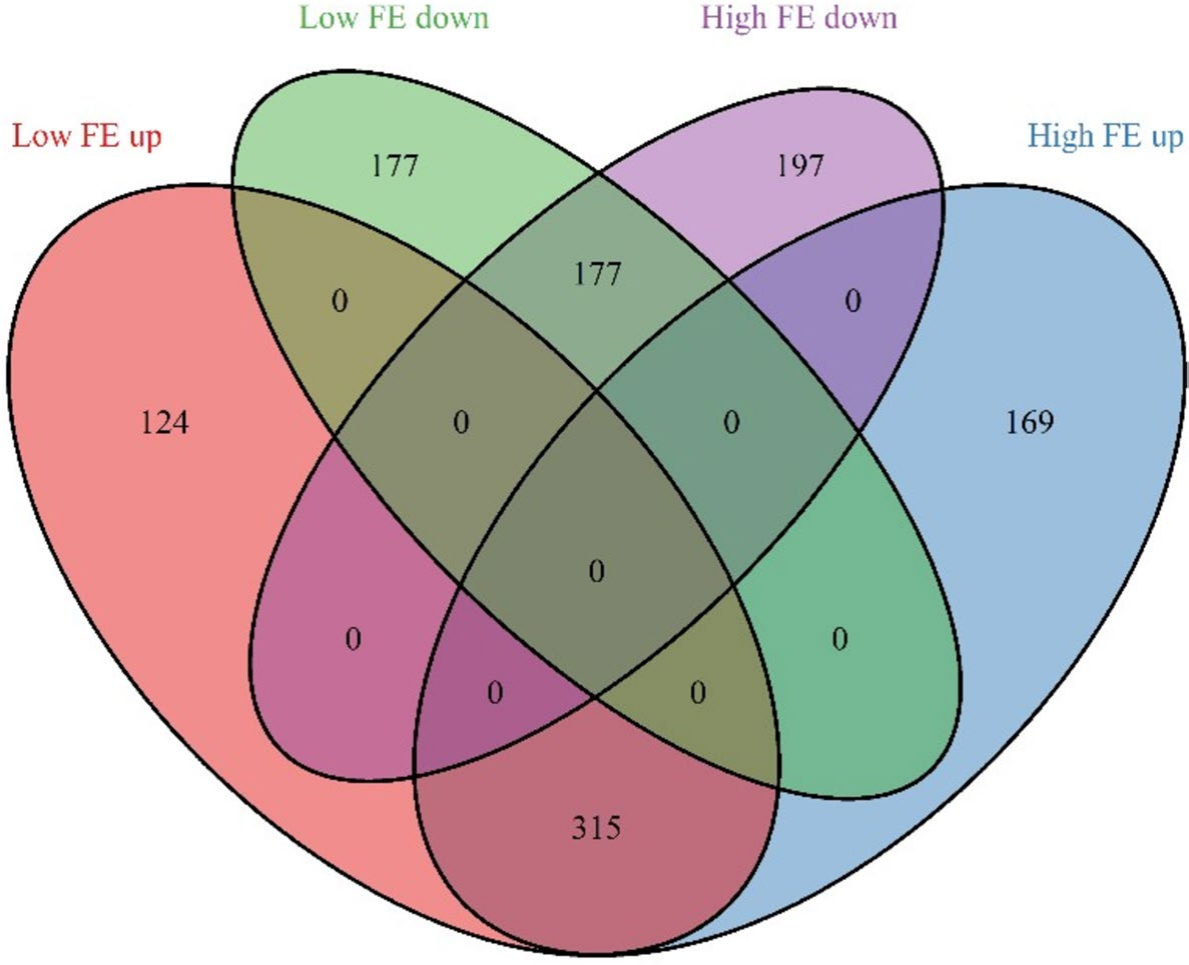
Venn diagram showing the numbers of genes that were significantly up or down regulated in colon organoids challenged with *E. coli* versus unchallenged colon organoids, in the high and low FE group, respectively. Low and High indicate the two FE groups and Up and Down indicate up or down regulated genes in the *E. coli* challenged organoids

The *E. coli* challenge had a large effect on gene expression profiles in both low and high FE organoids. A total of 1,159 genes were significantly regulated in response to the *E. coli* challenge in the low and/or high FE group (Fig. 4, Additional file 4). Of these, 492 were significant in both low and high FE organoids (315 upregulated and 177 downregulated in response to the *E. coli* challenge). Another 301 genes (124 up and 177 downregulated) were significant only in the low FE organoids, and 366 other genes (169 up and 197 down regulated) only in the high FE organoids. None of the 492 genes found regulated in both FE groups were upregulated in one FE group and downregulated in the other FE group. In fact, of all 1,159 genes differentially expressed in either or in both FE groups, there were only 3 that had opposite signs for the Log2 fold change (FC) in the two FE groups. This indicates a strong common response to the *E. coli* challenge regardless of the FE phenotype. Furthermore, the high and low FE colon organoids do not seem to differ substantially in range and average of the fold changes. In both groups there were more genes up regulated than down regulated. Also, generally the FC in expression of the upregulated genes was higher than that of the downregulated genes.

### Colon organoids functional enrichment response after an *E. coli* challenge

**Fig. 5:**
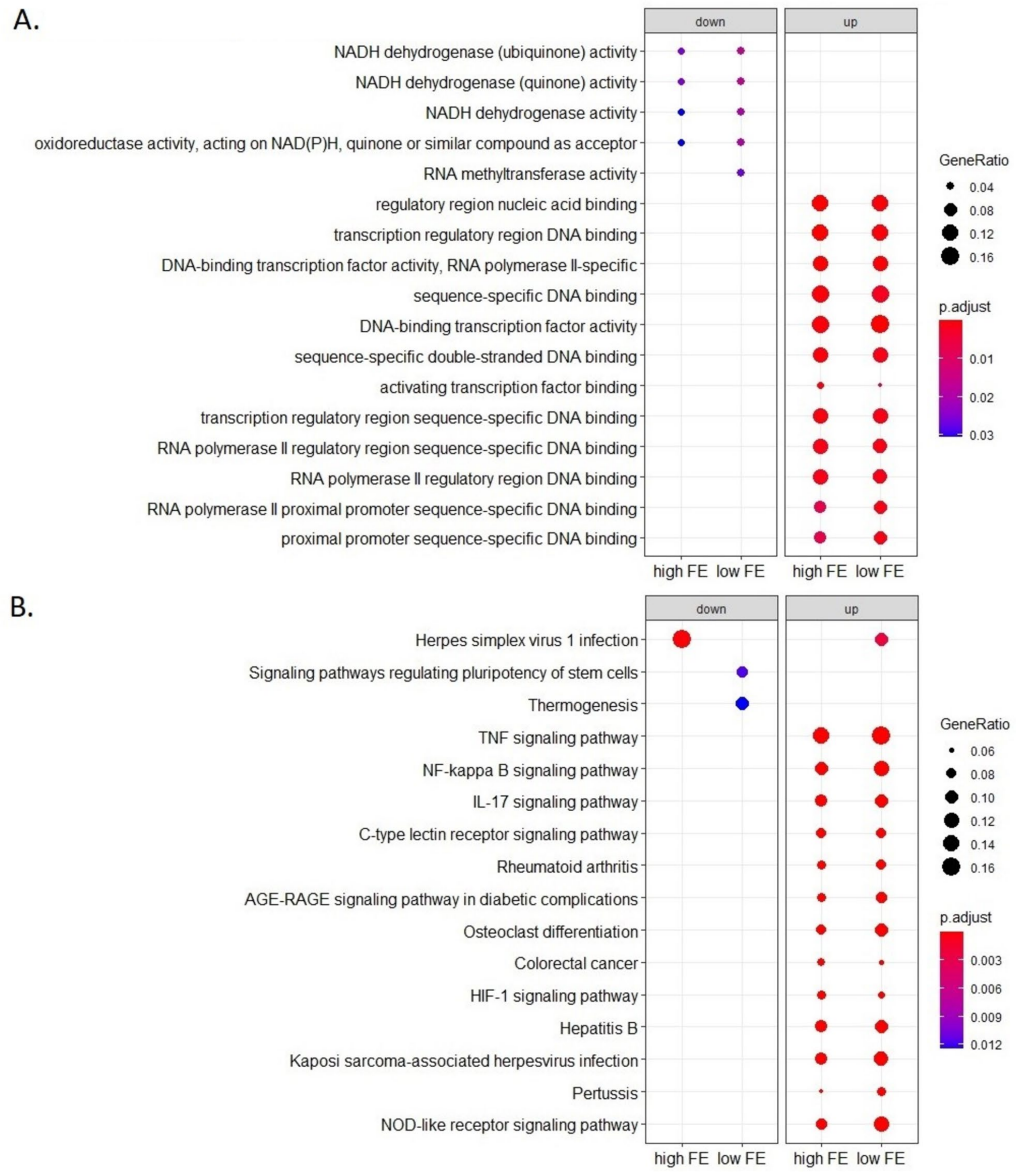
(**A**) Gene Ontology Biological Processes and (**B**) KEGG pathway analysis of the differentially expressed (challenged versus unchallenged) genes in low/high FE colon organoids. All DEGs were used in the enrichment analysis, i.e. the DEGs that were commonly found in both FE groups and the ones found uniquely in one of the FE groups

**Fig. 6:**
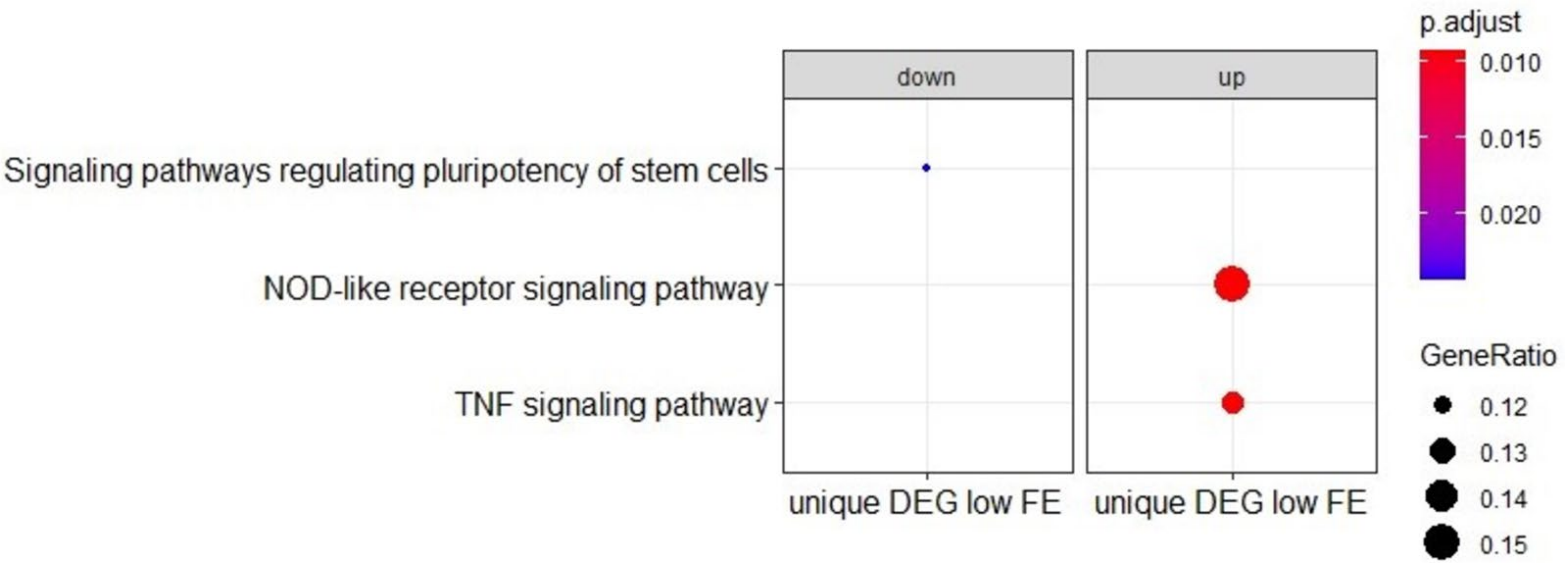
KEGG pathway analysis of the differentially expressed genes unique for the low FE group in challenged versus unchallenged colon organoids. All the DEGs are upregulated in the challenged group. Some genes could also be down regulated but that is not the case

Functional enrichment analysis (GO BP and KEGG) of the DEGs found in the comparison of challenged versus unchallenged colon organoids showed that high FE and low FE groups had in general similar Gene Ratio and adjusted p values for the observed GO BP and KEGG pathway enrichments (Fig. 5A and B). The GO enrichment analysis shows an up regulation of genes involved in gene expression; thus the *E. coli* challenge seems to stimulate the expression of genes. The KEGG pathway enrichment analysis showed a strong immune response for both high FE and low FE groups, but we did observe notable differences between the low and high FE groups in the KEGG enrichment analysis (Fig. 5B) with higher Gene Ratio and lower BH-adjusted p values for the TNF signalling pathway, NF-kappa B signalling pathway, IL17 signalling pathway, and NOD-like receptor signalling pathway in the low FE organoids (Table 2). These four immune related pathways are upregulated in both low and high FE colon organoids in response to the *E. coli* challenge, but more DEGs were identified in the low FE organoids resulting in stronger significant enrichments of these pathways (Table 2 and Additional files 7, 8, 9 and 10 (KEGG pathways figures)). Moreover, a KEGG functional enrichment analysis using only the 301 DEGs unique for low FE colon organoids in response to the *E. coli* challenge also resulted in enrichments of the TNF signaling pathway and NOD-like receptor signaling pathway (Fig. 6; Additional files 8 and 10). An enrichment analysis using the 366 DEGs unique for high FE organoids did not result in any functional enrichments. The strong representation of genes involved in immune signaling pathways was further underpinned by the fact that of the 20 genes with the highest fold change response to *E. coli* challenge (all upregulated), all genes, except for one (an uncharacterized gene), were clearly immune response associated genes, i.e. *TNF*, *CYP1A1*, *CXCL2*, *CCL20*, *TNFAIP2*, *RND1*, *NFKBIZ*, *CXCL8*, *TNFAIP3*, *CYP1B1*, *CSF2*, *NFKBIA*, *MAP3K8*, *AMCF-II*, *DDIT4*, *IL1A*, *IER3*, *ENSSSCG00000008954*, and *ENSSSCG00000031255* (Additional file 4). The latter two genes are uncharacterized genes but have been reported to be upregulated in alveolar macrophages in response to LPS [25]. In addition, the challenge of colon organoids with *E. coli* also resulted in significantly altered expression of 21 transporter genes, with notably strong upregulation (FC > 1) for *SLC5A3* (in high and low FE) and *SLC2A6* (significant in high FE) and with strong downregulation (FC <  − 1) for *SLC16A9* (significant in high and low FE), and *SLC6A5*, *SLC43A2*, *SLC26A4*, and *SCNN1G* (significant in low FE).

**Table 2:** Number of observed differentially expressed genes (# DEG) for low FE and high FE colon organoids as a response to the E. coli challenge

Pathway	Low FE Colon		High FE Colon	
	**# DEG**	**FDR**^**a**^	**# DEG**	**FDR**^**a**^
TNF signaling pathway (102)^a^	30	7.24E^−24^	25	2.44E^−17^
IL17 signaling pathway (84)^a^	19	5.39E^−13^	17	1.30E^−10^
NF-kappa B signaling pathway (92)^a^	22	1.74E^−14^	19	4.73E^−11^
NOD-like receptor signaling pathway (138)^a^	22	2.09E^−10^	16	5.52E^−06^

^a^Number between brackets is the number of total genes for each pathway and *FDR *False Discovery Rate. Pathways with indication of DEGs are shown in Additional files 7, 8, 9 and 10

### Descriptive statistics ileum organoids

**Fig. 7:**
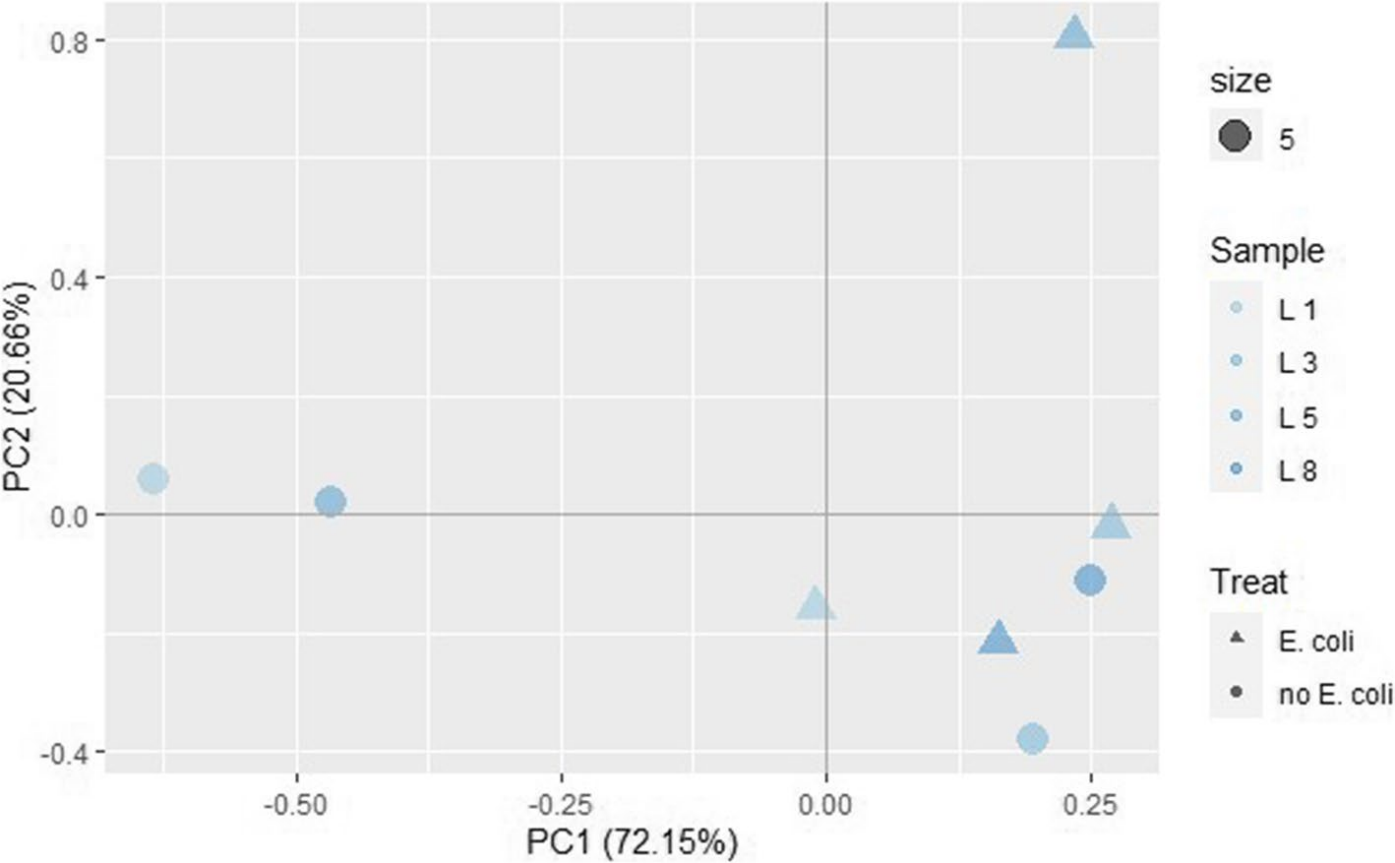
Principal component analysis plot of the ileum low FE organoid samples based on RSEM transcript per million (TPM) estimates (circles are unchallenged and triangles challenged Low FE organoids)

Whole genome RNA sequencing of eight ileum organoid samples (4 × low FE unchallenged and 4 × low FE *E. coli* challenged) resulted in 34,225,752 ± 5,505,957 (mean + SD) uniquely mapped reads, which was 95.29% ± 4.45% (mean ± SD) of the total number of reads after trimming (Additional file 5 for detailed alignment results of the ileum organoids). Clustering analysis based on global gene expression did not reveal a clear separation in the clustering between challenged versus unchallenged ileum samples, but for two samples (L1 and L5) the *E-coli* challenge seems to result in a large change in global gene expression (Fig. 7).

### Ileum specific genes

Of the 64 small intestine-specific genes retrieved from the TiGER database, 42 genes were expressed in the four low FE unchallenged ileum organoids (Additional file 6), suggesting that the ileum organoids have many of the similar cell types as the tissue they were derived from.

### Ileum organoid response to E. coli challenge

**Fig. 8:**
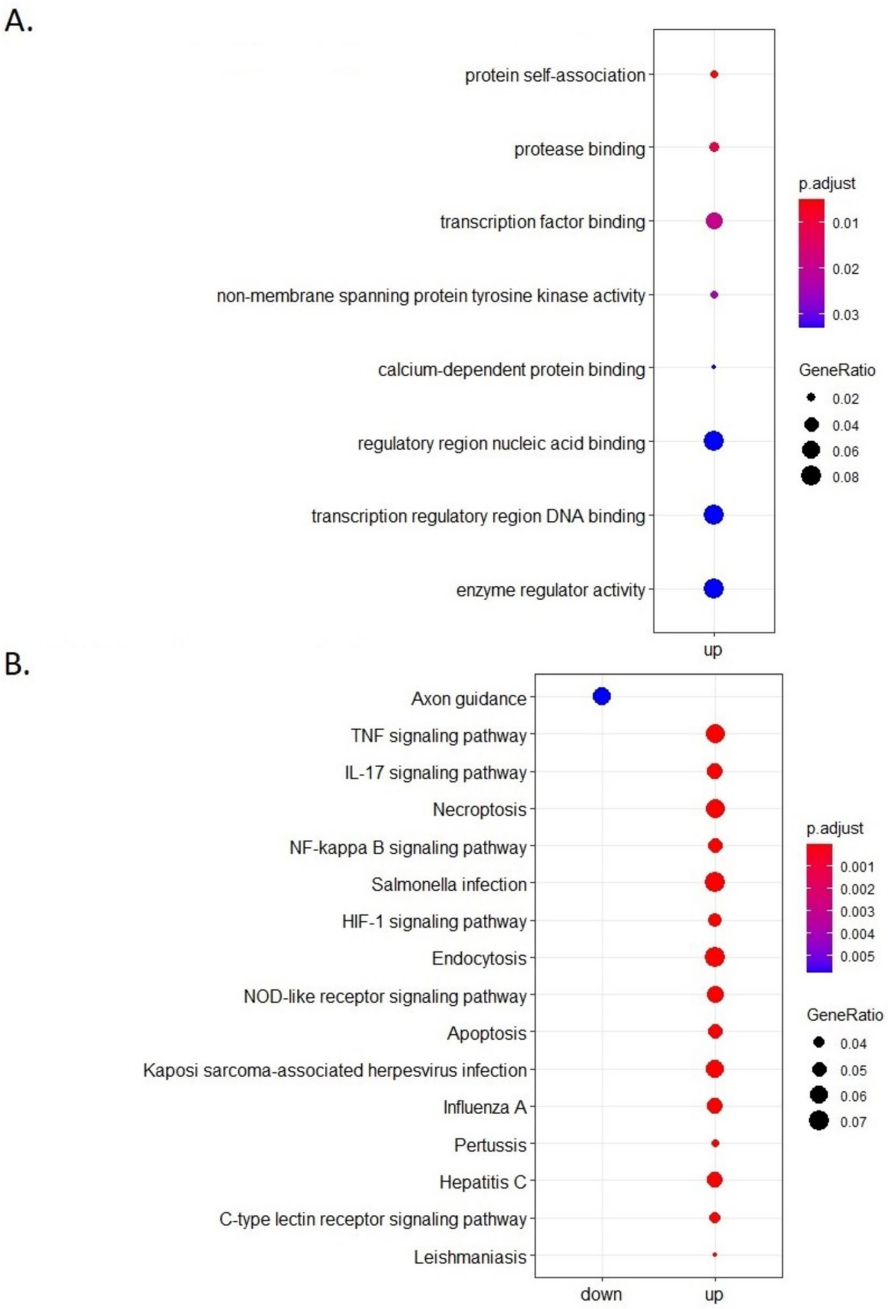
(**A**) Gene Ontology Biological Processes and (**B**) KEGG pathway analysis of the differentially expressed genes in challenged versus unchallenged low FE ileum organoids

For the ileum organoids, response to *E. coli* challenge was investigated for the low FE group using a GO BP and KEGG functional enrichment analysis (Fig. 8). The challenge with *E. coli* seems to have a stronger effect on gene expression profiles in ileum organoids (1974 DEGS) than in colon organoids. The GO BP analysis did not give an unambiguous signal whereas the KEGG enrichment analysis (Fig. 8B) clearly showed an immune response including the TNF signalling pathway, IL17 signalling pathway, NF-kappa B signalling pathway, and NOD-like receptor signalling pathway as enriched in DEGS upregulated in challenged versus unchallenged low FE ileum organoids.

### Comparison of low FE ileum and colon organoid enrichment analysis

Four immune related pathways were significantly enriched in DEGs (challenged versus unchallenged) in both the low FE colon and ileum organoids (Table 3). The number of DEGS in these pathways was higher in ileum than in colon organoids, suggesting a stronger immune-related response to *E. coli* in the ileum organoids (Additional files 7 and 8). More generally, the challenge with *E. coli* had a stronger effect on gene expression profiles in ileum organoids, as the challenge resulted in 1974 DEGs in (low FE) ileum organoids against 793 DEGs in (low FE) colon organoids.

**Table 3:** Number of observed differentially expressed genes (# DEG) for low FE colon and ileum organoids as a response to the E. coli challenge

Pathway	Low FE Colon		Low FE Ileum	
	**# DEG**	**FDR**^**a**^	**# DEG**	**FDR**^**a**^
TNF signalling pathway (102)^a^	30	7.24E^−24^	32	1.4E^−08^
IL17 signalling pathway (84)^a^	19	5.39E^−13^	27	1.1E^−07^
NF-kappa B signalling pathway (92)^a^	22	1.74E^−14^	24	2.0E^−05^
NOD-like receptor signalling pathway (138)^a^	22	2.09E^−10^	34	1.6E^−06^

^a^Number between brackets is number of total genes for each pathway and *FDR* False Discovery Rate. Pathways with indication of DEGs are shown in Additional files 7, 8, 9 and 10

## Discussion

The aim of this study was to explore the use of porcine intestinal organoids to study complex traits such as FE in pigs. The main findings are: 1) ileum and colon organoids expressed most of the tissue-specific genes of (the epithelial lining of) the respective tissue of origin, suggesting that many cell types of these tissues were represented in the respective intestinal organoids; 2) the two FE groups had only minor differences in colon organoid gene expression; 3) a challenge with *E. coli* resulted in strong gene expression changes in both ileum and colon organoids. Functional enrichment analyses of differential expressed genes indicated that the response was stronger in (low FE) ileum organoids than in (low FE) colon organoids; 4) the changes in expression of immune associated genes in response to the *E. coli* challenge was more pronounced in the low FE than in the high FE colon organoids.

We found only six genes differentially expressed between high FE versus low FE colon organoids (Fig. 3). Expression of *AMACR* was lower in high FE than in low FE pigs. Interestingly, lower expression of *AMACR* has also been reported in (faster growing, more efficient) Yorkshire pigs compared with Tibetan breeds [26]. This gene plays a role in the beta-oxidation of branched-chain fatty acids and fatty acid derivatives [27]. *ENSSSCG00000035617* and *HEBP1* were expressed stronger in high FE organoids than in low FE organoids. *ENSSSCG00000035617* was also seen upregulated in Yorkshire vs. Tibetan breed [26] and in enriched-housed pigs (which had increased growth rates) compared with barren-housed pigs [28], while *HEBP1* was reported to be clearly upregulated in colon of Ossabaw pigs given a ‘healthy’ diet compared with pigs given a ‘western’ diet [29].

*PRKD1* was expressed higher, and *RPL7a-like* and *PACSIN1* expressed lower, in the high FE vs. the low FE group. PRKD1 is known to have a wide range of intracellular functions (https://www.nextprot.org/entry/NX_Q15139/), but there is little known about PRKD1, RPL7a-like, and PACSIN1 in relation to colon or FE.

Vigors et al. [22] found 7 genes differentially expressed between unchallenged colon tissues from high and low FE piglets. In that study, *TRAM1* was just a bit lower expressed while *AOAH*, *AP1/JUN*, *TNF*, *IL10*, *CXCL8*, and *GPR43* were expressed higher in high FE vs. low FE pigs. Our results agreed with those of Vigors et al. [20, 22] in that we also measured upregulation of *AP1/JUN* (AP1 subunit genes), *TNF*, and *CXCL8* in response to *E. coli* challenge (see below). However, in our study, *IL-10* and *FFAR2* (*GPR43*) were not expressed and *AOAH* was hardly expressed in the colon organoids and expression of the other mentioned genes did not seem to differ at all between unchallenged high FE versus low FE colon organoids.

In the current study we have investigated the response of ileum and colon organoids to a challenge with LF82 adherent/invasive *E. coli*, and compared the response of the colon organoids from the two FE groups. The rationale for this is that it is well documented that immune responsiveness and inflammation in the animal can affect FE. An inflammation or infection can lead to decreased appetite and reduced feed intake [10–13], resulting in reduced growth. This means that a certain amount of accretion simply takes more time and therefore more maintenance energy, i.e. there is a larger expense per unit body gain. In addition, pro-inflammatory cytokines can lead to changing levels of circulating insulin, glucagon and corticosterone, associated with profound changes of intermediary metabolism with a shift from anabolism to catabolism [13]. This response to immunological stress does not necessarily serve to liberate energy and resources to be used by the immune system. In fact, comprehensive quantitative analyses of the costs of the immune system in human [30] and chicken [10] showed that the need for nutrients for supporting a resting immune system and for mounting an immune response during an infectious challenge is very small relative to resources used for growth. In contrast, the metabolic changes that occur as a result of the inflammation represent significant costs that, together with reduced feed intake and possibly the additional costs of fever, explain the depression of performance that is associated with inflammation and disease [10, 13].

In addition, inflammation can seriously compromise gut functionality and nutrient absorption [11, 18, 31]. Even low-grade inflammation may affect the health and total surface area of intestinal villi [14–17]. Inflammation also leads to an increased passage rate of digesta along the gastro-intestinal tract, reducing the time available for nutrients to be digested and absorbed [18]. Increased passage rate is often seen associated with (and may be the cause of) a high microbiota richness [32] and references therein.

In our study, both Ileum and colon organoids showed marked gene expression changes in response to the challenge with *E. coli*. A large number of genes and pathways/processes linked to immune-signaling were involved in the response. In colon organoids, the ‘top 20’ most strongly upregulated (highest FC) genes in response to *E. coli* challenge included 19 immune-related and immune-response associated genes *TNF*, *CYP1A1*, *CXCL2*, *CCL20*, *TNFAIP2*, *RND1*, *NFKBIZ*, *CXCL8*, *TNFAIP3*, *CYP1B1*, *CSF2*, *NFKBIA*, *MAP3K8*, *AMCF-II*, *DDIT4*, *IL1A*, *IER3*, plus *ENSSSCG00000008954* and *ENSSSCG00000031255* which both have been reported to be upregulated in alveolar macrophages in response to LPS [25]. Also Vigors and coworkers reported upregulation after LPS challenge of colon explants for immune-related genes *TNF*, *AP1/JUN*, *IL1*, *IL6*, *IL10*, *CXCL8*, *IFNG* and *SOCS3* [20, 22], which all, except *IL10* (not expressed in the organoids) and *SOCS3*, were also upregulated in challenged organoids in our study (albeit not significantly for *IL6*). Unfortunately we were not able to compare the two FE groups with regard to ileum organoids, but for the colon organoids, functional annotation analysis indicated a stronger response in low FE colon organoids than in high FE colon organoids in a number of immune signaling pathways, i.e. in pathways for TNF signaling, IL17 signaling, NF-kappa B signaling, and NOD-like receptor signaling. In the low FE group, the FDR values were clearly lower (and the number of DEGs identified in these four pathways appeared to be somewhat higher) than in the high FE group.

Challenge of colon organoids with *E. coli* also resulted in altered expression of a number of transporter genes. Five genes were downregulated quite strongly in challenged colon organoids: *SLC16A9*, *SLC6A5*, *SCNN1G*, *SLC26A4*, and *SLC43A2*. *SLC16A9* (*MCT9*), a proton-linked monocarboxylate transporter, was reported to be downregulated in ulcerative colitis [33]. SCNN1G and SLC26A4 transfer sodium ions and anions, respectively, and play a role in fluid and electrolyte homeostasis. Their downregulation may be involved in diarrhea [34]. Downregulation of *SLC43A2*, a transporter for neutral amino acids, may indicate a decreased focus on epithelial nutrient transport. However, this transporter is also implicated in immune function, as a too high expression (as seen in tumor cells) reduces the availability of methionine for T cells, and downregulation of *SLC43A2* can boost spontaneous and checkpoint-induced tumor immunity [35]. All five transporters appeared stronger downregulated in the low FE group (the latter four genes (*SLC6A5*, *SCNN1G*, *SLC26A4*, and *SLC43A2*) were significantly downregulated only in the low FE group), giving further support for a stronger immune response in the low FE colon organoids.

The number of animals in our study was not very large and the two groups did not differ very strongly in FE. Nevertheless, these results support other evidence that high FE pigs have a less pronounced response to infectious and non-infectious challenges, which could mitigate the ensuing changes of intermediary metabolism and gut functionality that generally result from inflammation. For instance, Vigors et al. [20] reported that pigs with low residual feed intake (RFI) (i.e. high FE) had consistently lower gene expression in the colon following an ex vivo challenge of jejunum and colon with LPS. Results from a later study from the same group [22] also indicated that LPS-induced up or down regulation of immune-related genes was less strong in high FE (low residual feed intake) than in low FE pigs for *IL1A*, *IL1B*, *IL10*, *IL8*, and *TLR1*, while it was similar in the two FE groups for the other genes that were seen to respond to LPS challenge in that study. Moreover, Vigors et al. [22] found that high FE pigs had higher expression of *AOAH*, an enzyme that can inactivate LPS, which could have contributed to the less avid response to LPS they observed in high FE pigs. Liu et al. [21] reported that low RFI had a relatively lower-level (but longer-lasting), inflammatory response after LPS injection and a lower rectal temperature. Also feed components can affect both immune functions and FE. Fiesel et al. [19] reported that pigs fed polyphenol-rich plant products had a higher FE and a lower expression of pro-inflammatory genes in duodenum, ileum and colon, Moreover, the high FE pigs had a lower feed intake and higher (less acidic) fecal pH, which suggests a slower passage of digesta. Similarly, Vigors et al. [23] found that high FE pigs had a lower feed intake, less acetate in colonic digesta, and higher populations of lactobacillus spp. in the cecum, which all suggest a slower passage of digesta [32, 36]. Furthermore, Vigors et al. [23] found that high FE pigs had increased apparent ileal digestibility of gross energy, and total tract digestibility of gross energy, nitrogen and dry matter. Also, they had higher relative gene expression in the jejunum of transporters and enzymes *FABP2*, *SGLT1*, *GLUT2*, and sucrase-isomaltase. As this enzyme and the transporters are markers for the brush border, this could indicate longer and/or healthier villi. Also Metzler-Zebeli et al. [24] reported that low RFI (high FE) pigs had shorter crypts, higher duodenal lactase and maltase activity and greater mucosal permeability, as well as lower basal expression of *TLR4* and *TNFA*.

Thus, the existing evidence suggests that a good balance between pro- and anti-inflammatory regulation in response to a challenge can be one of the factors explaining high FE, as a (too) avid immune response can negatively affect gut health and functionality and increase the costs of the metabolic changes and increased body temperature caused by inflammation. At the same time, it remains of course a necessity that an animal is able to have an adequate (but measured) immune response to prevent infections. It has been suggested that a strong genetic selection for FE could impair immune defense [21, 37–39], as indeed some immune related genes were reported to be expressed lower in high FE pigs [21, 24, 40]. However, in contrast, studies in pigs [21, 39, 41] and chicken [38, 42] indicated that animals with high FE can be robust and have an adequate or even better response to an infectious or noninfectious challenge than animals with low FE. For instance, Dunkelberger et al. [39] reported that high FE pigs were healthier, and were coping better with a PRRS challenge, having lower viral load and producing more antibodies, and growing better than less feed efficient pigs after PRRS challenge.

The current study has shown that organoids can be used to study specific molecular mechanisms related to FE. For instance, the expression of transporters can be studied. Even though intestinal organoids do not contain immune cells, they can be used to study the innate response to pathogens or to pathogen associated molecular patterns (PAMPs). If indeed, the magnitude of the immune response in the intact animal would affect its gut and villus health and functionality and would also increase the costs of inflammation, the immune-related responses measured in organoids may be a proxy for these important factors of FE in the animal.

## Conclusion

Organoids are a good representation of the organ they originate from. We identified differences in colon organoid gene expression between high FE and low FE pigs and in the innate immune response of low FE and high FE colon organoids challenged with *E. coli*. These findings show that organoids can be used to gain insights into complex biological mechanisms such as FE.

## Methods

### Animal material

The animal material used in this study originates from a three-way crossbreeding. In total seven ‘synthetic’ sires (S) and twelve sows (F1 Landrace (LR) x Large White (LW) crossbred) produced the growing-finishing piglets (S (LR x LW). The animals were kept in pens with 60% concrete floor and 40% slatted floor and pens were equipped with IVOG stations [43] at Mantinge, the Netherlands. Out of 40 piglets, twelve were selected with divergent phenotypes for FE. Six piglets with low feed conversion ratio (FCR) (mean 2.19 ± 0.03) were allocated to the “high FE group”, and six piglets with high FCR (mean 2.61 ± 0.04) were allocated to the “low FE group”. Piglets were slaughtered in a commercial slaughterhouse, under commercial conditions, approximately six months after birth and tissue from the ileum (~ 50 cm from ileocecal valve) and the proximal colon were collected. All piglets were males (boars).

### Organoid culture

Colon organoids were generated from intestinal tissue of two 6 month-old slaughter pigs, according to the procedure described by Sato and colleagues [44]. Porcine colon organoids were grown in basal culture medium (BCM) that was refreshed every two days (BCM: DMEM/F12 (Gibco), supplemented with 100 μg/ml primocin (Invivogen), 10 mM HEPES (HyClone), 1 × B-27 (Gibco), 1.25 mM N-acetylcysteine (Sigma), 50 ng/ml human epidermal growth factor (R&D systems), 15 nM gastrin, 10 mM nicotinamide, 10 μM p38 MAPK inhibitor (Sigma), 600 nM TGFβ receptor inhibitor A83-01, and (50% v/v) conditioned L-WRN medium prepared from L-WRN cells (ATCC®; Cat.# CRL-3276™) as previously described [45]. Organoids were passaged at a 1:5 ratio every 5 days by mechanical dissociation and plating in fresh Matrigel matrix droplets (Basement Membrane, Growth factor reduced, REF 356231, Corning, Bedford, MA, USA).

### Two-dimensional (2D) monolayers of 3D organoid cultures

2D monolayers of 3D colon organoids were prepared according to the method described in van der Hee et al. [3]. Briefly, colon organoid cells were recovered from several Matrigel droplets after 5 days growth by addition of ice-cold DMEM/F12 medium, and transfer into 15 ml tubes followed by centrifugation at 250 × g for 5 min. The pellet of organoids was then incubated in TrypLE Express dissociation medium (Gibco) for 10 min at 37 °C and dissociated by repeated pipetting to obtain a single cell suspension. Four volumes of BCM, enhanced with 20% (v/v) FBS (E-BCM) was added to the single cell suspension and centrifuged at 900 × g for 5 min. Cell pellets were resuspended in E-BCM, counted manually using a Bürker chamber and seeded at approximately 78,000 cells/cm2 in pre-coated culture plates or Transwells. The pre-coating procedure involved incubation with 0.5% (v/v) Matrigel in F12 medium at 37 °C for 1 h after which the liquid was removed, and the plates were air-dried for 10 min. After 3 days incubation at 37 °C (5% CO_2_) the cell monolayers reached confluence and were used for experiments.

### Experimental design

**Fig. 9:**
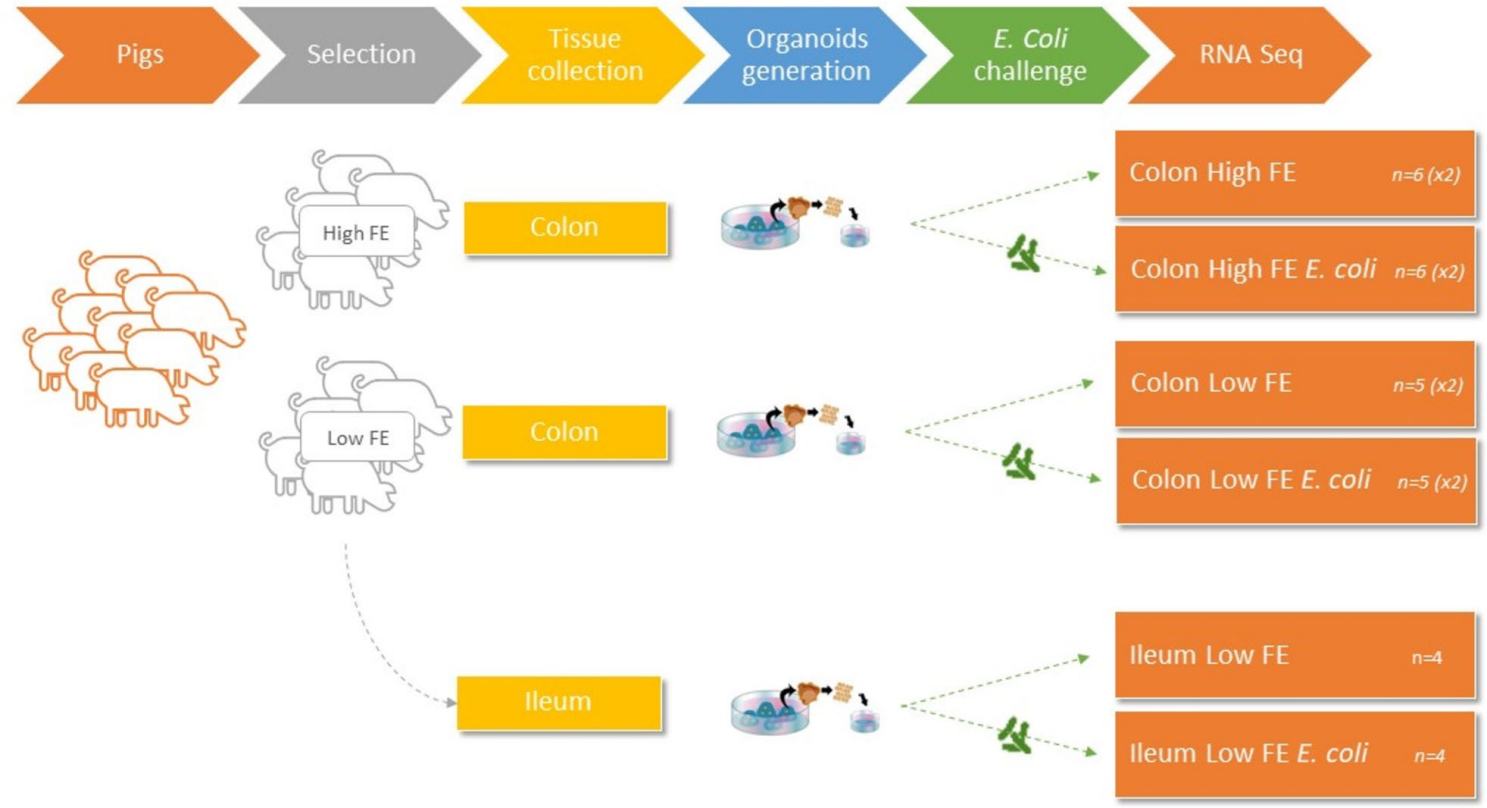
Experimental design. Pigs were selected for a low or high feed efficient (FE) phenotype. From each piglet, 4 colon and 2 ileum tissue samples were taken to generate replicate 3D organoid cultures, from which 2D organoids were produced. Gene expression was measured after incubation of 2D organoids during 5 h with or without the presence of LF82 adherent/invasive *E. coli* (challenged/control). See Table 4 for number of pigs and replicates per FE group, per tissue, per treatment

A visualization of the experimental design is given in Fig. 9. In both FE groups, per piglet, 4 colon and 2 ileum tissue samples were taken to produce replicate 3D organoid cultures. Colon organoids were obtained from six high FE and five low FE piglets. However, due to a contamination, ileum organoids were only obtained from 4 low FE piglets. Two-dimensional (2D) organoids were derived from the 3D organoid cultures and gene expression was measured after incubation of the 2D organoids during 5 h with or without the presence of LF82 adherent/invasive *E. coli* (challenged/control). See Table 4 and Fig. 9 for number of pigs and replicates per FE group, per tissue, per treatment.

**Table 4:** Number of pigs and replicates per FE group, per tissue, per treatment

Group	organ	# pigs	# samples per pig per tissue	2D Organoids per pig without *E. coli*	2D Organoids per pig with *E. coli*
High FE	Colon	6	4	2	2
Low FE	Colon	5	4	2	2
Low FE	Ileum	4	2	1	1

### RNA sequencing

Total RNA was isolated from organoids using the Qiagen RNeasy Mini Kit following manufacturer’s protocol and quantified using a NanoDrop spectrophotometer. RNA sequencing was done at Novogene with the Illumina TruSeq RNA sample protocol, producing approximately 30,000,000 paired end stranded reads of 150 bp for each sample (Additional files 1 and 5). Quality of the raw sequencing data were accessed with FastQC (v0.11.7) [46]. Trim Galore (v0.5.0) [47] with Cutadapt (v1.16) [48] and default settings except from -l 6 (stringency of 6 bp), was used to trim low-quality data and to remove the Illumina sequencing adaptors, poly A tails and keeping only paired-end reads if both reads were ≥ 35 bp.

### Alignment, expression quantification and differential expression analysis

Trimmed reads were aligned against the pig reference genome (Ensembl Sus scrofa 11.1.93) [49] using STAR version 2.7 with default settings [50]. RSEM v1.3.1 [51] was used to quantify gene expression with default settings, except for the strand specific protocol, which was set to 0 to derive all upstream reads from the reverse strand. RSEM expected counts and Transcript Per Million (TPM) values were quantified. TPM values were used to determine the expression of colon and ileum specific genes with TPM > 1 as a threshold of gene expression and expected counts were used in downstream analysis to determine Differentially Expressed Genes (DEGs). Two R packages were used for DEG analysis in R version 3.5.3 (R Development Core Team, 2019). 1) DESeq2 version 1.22.2 [52] where RSEM expected counts were imported via the recommended pipeline (DESeqDataSetFromTximport), applying default normalization for sequencing depth [53]. 2) EdgeR version 3.24.3 [54] where RSEM expected counts were imported as a count matrix (gene_i_ x sample_j_). To compare DEG output of EdgeR and DESeq2, a comparable normalization procedure in EdgeR was used (“*Relative Log Expression*”). For both packages a False Discovery Rate (FDR) < 0.05 (adjusted p-value for multiple testing according to the Benjamini–Hochberg correction) [55] were used as a threshold to identify significant DEGs. Downstream analyses focused on the overlapping DEGs found by both programs, to reduce false positives. A visualization of the experimental and analytics of this study is given in Fig. 2.

### Colon and ileum specific genes

To investigate whether the organoids resemble the tissue they are derived from (ileum or colon), gene expression of the unchallenged organoids was compared to a reference list of genes that are commonly expressed in the colon and small intestine, respectively, obtained from the Tissue-specific Gene Expression and Regulation (TiGER) database [56]. Additionally, to determine the usability of the colon organoids results, genes that previously have been suggested to be related with FE and immune challenges from studies on tissues were tested for expression in the unchallenged colon organoids [20, 22].

### Gene enrichment analysis

Gene Ontology Biological Processes (GO BP) [57, 58] and Kyoto Encyclopedia of Genes and Genomes (KEGG) [59] functional enrichment analyses were performed by R package ClusterProfiler [60]. For the KEGG analysis the ENSEMBL gene identifiers were converted to NCBI gene identifiers via the ENSEMBL Biomart data mining tool [61]. To prevent a high FDR due to multiple testing, the Benjamini & Hochberg FDR correction was used to adjust *p*-values in both GO and KEGG enrichment analysis. FDR < 0.05 was chosen as the threshold.

R package limma (version 3.40.6) [62] was used to plot the distances between the samples, gplots (version 3.0.3) [63] to create heatmaps of the gene expression profiles and VennDiagram (version 1.6.20) [64] to create Venn diagrams.

### Supplementary Information

**Additional file 1. **RNA-seq data and alignment results of the 44 colon organoids samples.

**Additional file 2. **Colon specific genes retrieved from TiGER database, mean and standard deviation (SD) of RSEM TPM values for 22 unchallenged low and high FE colon organoid samples. Red indicates genes not expressed (Threshold: TPM < 1).

**Additional file 3. **The DESeg2 and EdgeR results of the differentially expressed genes (DEGs) between unchallenged low and high FE organoids (FDR = False Discovery Rate).

**Additional file 4. **Table with expression and statics from DESeq2 and edgeR analyses of significantly differential expressed genes after e-coli challenge in colon (high and low FE) and ileum (low FE).

**Additional file 5. **RNA-seq data and alignment results of the 8 ileum organoid samples.

**Additional file 6. **Small intestine specific genes retrieved from TiGER database, mean and standard deviation (SD) of RSEM TPM values for 4 unchallenged low FE ileum organoid samples. Red indicate genes not expressed (Threshold: TPM < 1).

**Additional file 7. **DEGs (indicated in red) in the IL17 signaling KEEG pathway between unchallenged and challenged colon high (top), colon low (middle) and ileum low (lowest) organoids. Permission for the use of these figures was obtained from KEGG.

**Additional file 8. **DEGs (indicated in red) in the TNF signaling KEGG pathway between unchallenged and challenged colon high (top), colon low (middle) and ileum low (lowest) organoids. Permission for the use of these figures was obtained from KEGG.

**Additional file 9. **DEGs (indicated in red) in the NF-kappa B signaling KEEG pathway between unchallenged and challenged colon high (top), colon low (middle) and ileum low (lowest) organoids. Permission for the use of these figures was obtained from KEGG.

**Additional file 10. **DEGs (indicated in red) in the NOD signaling KEGG pathway between unchallenged and challenged colon high (top), colon low (middle) and ileum low (lowest) organoids. Permission for the use of these figures was obtained from KEGG.

## Data Availability

The RNA-seq data describe in this study is available by the following SRA/ENA accession PRJEB57598 (https://www.ebi.ac.uk/ena/browser/view/PRJEB57598).

## Abbreviations

2D: Two dimensional
3D: Three dimensional
DEGs: Differentially expressed genes
*E. coli*: *Escherichia coli*
FC: Fold change
FCR: Feed conversion ratio
FDR: False discovery rate
FE: Feed efficiency
GO: Gene Ontology
GO BP: Gene Ontology Biological Processes
KEGG: Kyoto Encyclopedia of Genes and Genomes
LPS: Lipopolysaccharide
LR: Landrace
LW: Large White
PAMPs: Pathogen associated molecular patterns
RFI: Residual feed intake
S: ‘synthetic’ sires
SD: Standard deviation
TiGER: Tissue-specific Gene Expression and Regulation
TPM: Transcript Per Million
