# Supplementary material for: Transcriptomic analysis of intestinal organoids, derived from pigs divergent in feed efficiency, and their response to Escherichia coli
Source: BMC Genomics. 2024 Feb 13;25:173. doi: 10.1186/s12864-024-10064-0 (PMC10863143; doi:10.1186/s12864-024-10064-0)
Supplement: Supplementary file 1 — Additional file 1. RNA-seq data and alignment results of the 44 colon organoids samples. [file 12864_2024_10064_MOESM1_ESM.pdf]

Additional file 1. RNA-seq data and alignment results of the 44 colon organoids samples

Group	Sample	Replicate	Raw reads	Trimmed reads	Uniquely mapped reads	% Uniquely mapped reads
**Low**	**2**	**1**	33,789,331	33,374,900	32,022,491	95.95
		**2**	35,726,364	35,309,894	33,753,802	95.59
	**3**	**1**	35,635,910	35,196,607	33,479,344	95.12
		**2**	31,051,327	30,677,225	29,017,206	94.59
	**5**	**1**	30,956,870	30,564,649	29,239,187	95.66
		**2**	33,801,922	33,402,924	31,878,411	95.44
	**8**	**1**	31,015,974	30,670,015	29,499,096	96.18
		**2**	38,696,781	38,232,659	36,424,517	95.27
	**11**	**1**	36,434,997	36,006,713	34,370,505	95.46
		**2**	30,594,442	30,251,394	28,659,848	94.74
**Low**	**2**	**1**	33,848,722	33,481,244	32,225,047	96.25
E. coli		**2**	35,407,139	34,970,938	33,387,923	95.47
	**3**	**1**	35,606,275	35,163,069	33,491,497	95.25
		**2**	32,628,560	32,228,384	30,487,013	94.60
	**5**	**1**	31,137,837	30,745,870	29,381,049	95.56
		**2**	33,328,899	32,904,484	31,326,619	95.20
	**8**	**1**	32,717,625	32,327,982	30,994,841	95.88
		**2**	32,214,141	31,817,235	30,296,880	95.22
	**11**	**1**	35,160,709	34,730,585	33,151,772	95.45
		**2**	32,843,986	32,467,464	30,748,181	94.70
**High**	**4**	**1**	33,003,772	32,564,498	31,084,129	95.45
		**2**	32,604,835	32,225,072	30,710,371	95.30
	**6**	**1**	37,211,770	36,775,210	35,146,161	95.57
		**2**	33,598,668	33,199,038	31,569,110	95.09
	**7**	**1**	34,233,346	33,852,458	32,130,709	94.91
		**2**	30,516,726	30,172,430	28,410,558	94.16
	**9**	**1**	34,975,883	34,590,031	33,173,779	95.91
		**2**	31,936,840	31,572,514	30,238,990	95.78
	**10**	**1**	32,035,675	31,637,030	30,042,678	94.96
		**2**	35,938,549	35,530,857	33,595,052	94.55
	**12**	**1**	31,063,240	30,687,599	29,523,271	96.21
		**2**	32,966,166	32,581,343	31,129,043	95.54
**High**	**4**	**1**	35,057,765	34,624,226	33,140,915	95.72
E. coli		**2**	30,924,959	30,524,631	29,033,116	95.11
	**6**	**1**	30,264,439	29,884,710	28,597,366	95.69
		**2**	34,006,935	33,552,783	31,957,908	95.25
	**7**	**1**	34,684,129	34,259,759	32,541,159	94.98
		**2**	34,359,108	33,954,646	31,918,312	94.00
	**9**	**1**	33,310,381	32,915,816	31,560,137	95.88
		**2**	40,119,142	39,614,239	37,770,353	95.35
	**10**	**1**	31,924,422	31,552,594	29,862,819	94.64
		**2**	32,675,396	32,266,732	30,447,214	94.36
	**12**	**1**	36,269,018	35,848,164	34,362,925	95.86
		**2**	37,416,473	36,973,407	35,325,470	95.54
