# Supplementary material for: Transcriptomic analysis of intestinal organoids, derived from pigs divergent in feed efficiency, and their response to Escherichia coli
Source: BMC Genomics. 2024 Feb 13;25:173. doi: 10.1186/s12864-024-10064-0 (PMC10863143; doi:10.1186/s12864-024-10064-0)
Supplement: Supplementary file 2 — Additional file 2. Colon specific genes retrieved from TiGER database, mean and standard deviation (SD) of RSEM TPM values for 22 unchallenged low and high FE colon organoid samples. Red indicates genes not expressed (Threshold: TPM < 1). [file 12864_2024_10064_MOESM2_ESM.pdf]

Additional file 2. Colon specific genes retrieved from TiGER database, mean and standard deviation (SD) of RSEM TPM values for 22 unchallenged low and high FE colon organoid samples. Red indicates genes not expressed (Threshold: TPM < 1)

Genes	mean	SD	Genes	mean	SD	Genes	mean	SD
A2ML1	0.06	0.04	*GPA33*	178.30	82.59	*PSEN1*	52.15	12.80
ADAMTS13	0.47	0.67	*GPR35*	7.70	4.95	*PTPRH*	23.09	23.38
ANXA13	35.01	24.46	*GPX2*	241.63	116.57	*R3HDML*	0.01	0.03
APOBEC1	69.14	33.96	*GRM8*	0.00	0.01	*RAB20*	7.73	3.15
AQP8	0.18	0.29	*HEPH*	138.82	64.10	*REG4*	579.67	336.38
ARHGAP25	0.02	0.04	*HKDC1*	1.46	0.53	*REN*	0.00	0.01
ASCL2	0.29	0.34	*HNF4A*	97.08	43.17	*RNF186*	1.31	2.48
AXIN2	6.56	1.24	*HOXB6*	28.80	11.02	*RSPO1*	0.00	0.01
B3GNT3	251.78	62.25	*HOXB8*	5.00	1.50	*SATB2*	45.05	9.43
BAIAP2L2	41.92	40.78	*HOXB9*	12.92	2.63	*SENP5*	10.26	1.50
BMP4	27.13	5.63	*IHH*	47.15	35.21	*SGK2*	17.03	14.76
CDH17	587.78	391.52	*IL1RL2*	0.00	0.00	*SIM2*	0.06	0.07
CDX1	141.96	36.65	*IL22RA1*	28.75	17.25	*SLC12A2*	108.02	44.72
CDX2	16.12	10.50	*KCNH8*	0.00	0.01	*SLC17A4*	0.00	0.00
CFTR	7.02	4.52	*KCNQ1*	241.75	32.89	*SLC22A18*	33.89	8.40
CLCA1	36.18	30.19	*KRT20*	34.96	29.13	*SLC26A3*	238.56	243.32
CLCA4	437.43	369.01	*LGALS4*	310.45	95.93	*SLC44A3*	17.73	4.92
CLDN2	0.97	0.61	*LIPH*	186.40	52.09	*SLC5A1*	8.04	2.78
CLDN3	441.42	214.51	*LRP4*	0.01	0.01	*SLC6A20*	1.50	1.48
COL8A2	0.07	0.03	*MLXIPL*	0.97	0.79	*SLC9A2*	2.51	2.50
CORIN	0.09	0.08	*MST1R*	156.24	27.01	*ST14*	337.96	40.71
CYP2S1	9.39	7.13	*MT1A*	39.53	37.99	*TFF3*	158.11	172.48
DAPK2	0.26	0.31	*MUC13*	179.32	105.19	*TMC5*	7.09	4.46
DNMT3A	5.57	0.71	*MYH14*	157.45	65.11	*TMEM45B*	146.92	79.37
DPEP1	1.09	1.37	*MYO1A*	22.46	24.57	*TMEM54*	302.89	131.18
DUOX2	0.23	0.21	*MYO7B*	159.39	53.42	*TMEM92*	8.61	6.54
EDAR	2.33	1.30	*OLFM4*	0.04	0.07	*TMPRSS3*	0.13	0.16
EPHB3	6.44	2.07	*OVOL2*	2.99	1.13	*TMPRSS4*	13.53	11.92
ETV3	80.05	13.16	*PIGR*	7.02	3.75	*TRIM15*	3.56	2.55
EVX1	0.54	0.32	*PKP2*	109.09	19.43	*TRIM7*	1.02	0.53
FANCB	1.03	0.31	*PLEKHG6*	24.98	11.26	*TSPAN1*	2499.41	1777.71
FOXA3	0.87	0.31	*POF1B*	11.46	11.72	*USH1C*	36.21	14.79
FREM2	0.10	0.05	*POU2F3*	0.44	0.34	*VIL1*	351.42	138.74
GCNT3	42.00	38.92	*PPP1R14D*	73.32	40.23	*XKR9*	0.95	0.29
GMDS	68.25	7.39	*PROM2*	66.69	29.62			
