# Supplementary material for: Transcriptomic analysis of intestinal organoids, derived from pigs divergent in feed efficiency, and their response to Escherichia coli
Source: BMC Genomics. 2024 Feb 13;25:173. doi: 10.1186/s12864-024-10064-0 (PMC10863143; doi:10.1186/s12864-024-10064-0)
Supplement: Supplementary file 3 — Additional file 3. The DESeg2 and EdgeR results of the differentially expressed genes (DEGs) between unchallenged low and high FE organoids (FDR = False Discovery Rate). [file 12864_2024_10064_MOESM3_ESM.pdf]

Additional file 3. The DESeg2 and EdgeR results of the differentially expressed genes (DEGs) between unchallenged low and high FE organoids (FDR = False Discovery Rate).

ENSEMBL ID	Gene Name	DESeq2		EdgeR	
		logFC	FDR	logFC	FDR
ENSSSCG00000027053	*PACSIN1*	1.662	6.34E-07	1.669	2.27E-06
ENSSSCG00000040275	*HEBP1*	-0.524	6.34E-07	-0.527	6.75E-05
ENSSSCG00000016822	*AMACR*	1.358	0.0002	1.358	0.0002
ENSSSCG00000035617	*RPL7a-like*	-1.036	0.0015	-1.046	0.0056
ENSSSCG00000001975	*PRKD1*	-2.273	0.0010	-2.223	0.0179
ENSSSCG00000022842	.	1.010	0.0211	1.006	0.0405
