# Supplementary material for: Transcriptomic analysis of intestinal organoids, derived from pigs divergent in feed efficiency, and their response to Escherichia coli
Source: BMC Genomics. 2024 Feb 13;25:173. doi: 10.1186/s12864-024-10064-0 (PMC10863143; doi:10.1186/s12864-024-10064-0)
Supplement: Supplementary file 5 — Additional file 5. RNA-seq data and alignment results of the 8 ileum organoid samples. [file 12864_2024_10064_MOESM5_ESM.pdf]

Additional file 5. RNA-seq data and alignment results of the 8 ileum organoid samples

Group	Sample	Raw reads	Trimmed reads	Uniquely mapped reads	% Uniquely mapped reads
**Low**	**1**	47,287,772	46,643,676	45,149,918	96.80
	**3**	33,617,465	33,203,932	31,881,142	96.02
	**5**	42,755,196	42,157,941	40,978,048	97.20
	**8**	33,478,864	33,123,448	27,680,830	83.57
**Low**	**1**	32,457,431	32,065,030	31,187,090	97.26
E. coli	**3**	36,027,309	35,594,324	34,596,780	97.20
	**5**	31,802,560	31,401,004	30,402,444	96.82
	**8**	33,169,307	32,772,650	31,929,766	97.43
