# Supplementary material for: Transcriptomic analysis of intestinal organoids, derived from pigs divergent in feed efficiency, and their response to Escherichia coli
Source: BMC Genomics. 2024 Feb 13;25:173. doi: 10.1186/s12864-024-10064-0 (PMC10863143; doi:10.1186/s12864-024-10064-0)
Supplement: Supplementary file 6 — Additional file 6. Small intestine specific genes retrieved from TiGER database, mean and standard deviation (SD) of RSEM TPM values for 4 unchallenged low FE ileum organoid samples. Red indicate genes not expressed (Threshold: TPM < 1). [file 12864_2024_10064_MOESM6_ESM.pdf]

Additional file 6. Small intestine specific genes retrieved from TiGER database, mean and standard deviation (SD) of RSEM TPM values for 4 unchallenged low FE ileum organoid samples. Red indicate genes not expressed (Threshold: TPM < 1)

Genes	mean	SD	Genes	mean	SD
ABCA3	4.82	1.66	*M6PR*	123.03	18.73
ALDH1A1	506.12	330.62	*MEP1B*	0.05	0.08
ALDOB	0.24	0.63	*MMRN1*	0.00	0.00
APOB	3.11	4.91	*MTTP*	0.97	0.88
BMP4	24.68	12.41	*NDST1*	26.89	6.75
C15orf39	14.68	2.09	*NELL2*	0.05	0.06
CALR	734.26	88.75	*OSR2*	33.28	16.54
CCDC9	30.89	2.58	*PAX6*	0.61	1.18
CDH17	27.13	44.09	*PCDH18*	0.74	0.50
CLCA1	329.84	545.16	*PCDH9*	0.05	0.10
CLCN7	16.69	3.41	*PHF6*	18.33	2.20
CLPTM1	84.49	9.78	*PTDSS2*	11.52	1.09
CNNM2	0.20	0.09	*SCD*	72.59	23.88
COL5A2	0.43	0.14	*SEC14L1*	35.66	5.53
CPT1C	0.06	0.07	*SEC61A1*	119.29	23.17
DHCR7	50.11	5.31	*SI*	0.00	0.00
DKK1	0.00	0.00	*SLC26A3*	0.58	0.84
DSC2	104.66	45.89	*SLC39A1*	73.62	4.76
DUSP6	34.66	7.66	*SLC4A2*	73.96	16.01
EDNRA	0.91	0.86	*SLIT2*	0.38	0.33
ENC1	131.69	41.98	*SNCA*	0.08	0.10
EPHA7	0.08	0.08	*SPCS2*	158.86	12.37
FGF10	0.00	0.00	*SPIN1*	52.65	9.25
FURIN	98.55	24.57	*SPRY2*	29.18	11.16
GAA	26.78	1.99	*STX12*	106.38	10.27
GANAB	214.00	15.74	*SYPL2*	0.18	0.17
GCG	0.03	0.04	*TCOF1*	24.76	6.93
IPO4	49.14	4.88	*TFRC*	77.45	24.87
ITGAV	196.59	61.34	*TNFRSF19*	2.11	1.26
KIF1A	0.28	0.14	*TPBG*	28.97	6.77
KITLG	50.77	16.41	*ZNF398*	5.36	0.83
KREMEN2	3.80	1.38	*ZNF512*	3.23	1.27
