# Supplementary material for: Transcriptomic analysis of intestinal organoids, derived from pigs divergent in feed efficiency, and their response to Escherichia coli
Source: BMC Genomics. 2024 Feb 13;25:173. doi: 10.1186/s12864-024-10064-0 (PMC10863143; doi:10.1186/s12864-024-10064-0)
Supplement: Supplementary file 7 — Additional file 7. DEGs (indicated in red) in the IL17 signaling KEEG pathway between unchallenged and challenged colon high (top), colon low (middle) and ileum low (lowest) organoids. Permission for the use of these figures was obtained from KEGG. [file 12864_2024_10064_MOESM7_ESM.pdf]

Additional file 7: DEGs (indicated in red) in the IL17 signaling KEEG pathway between unchallenged and challenged colon high (top), colon low (middle) and ileum low (lowest) organoids. Permission for the use of these figures was obtained from KEGG.

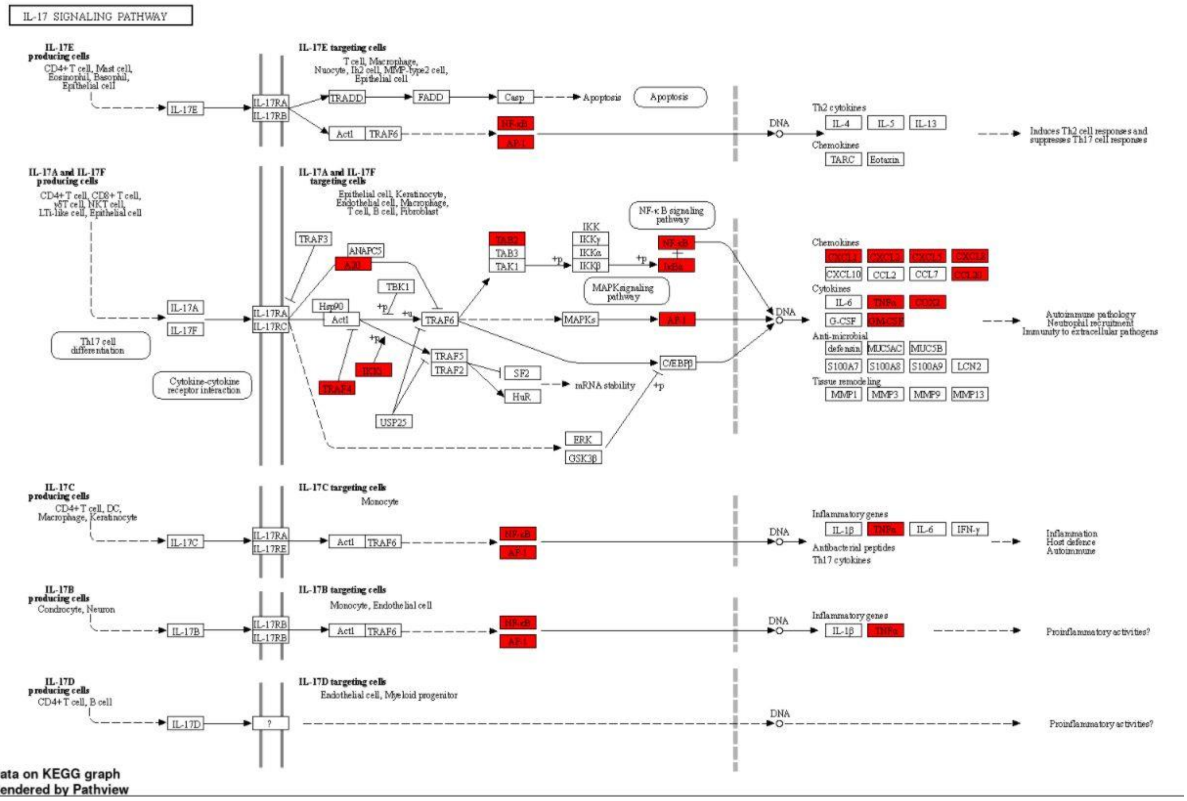

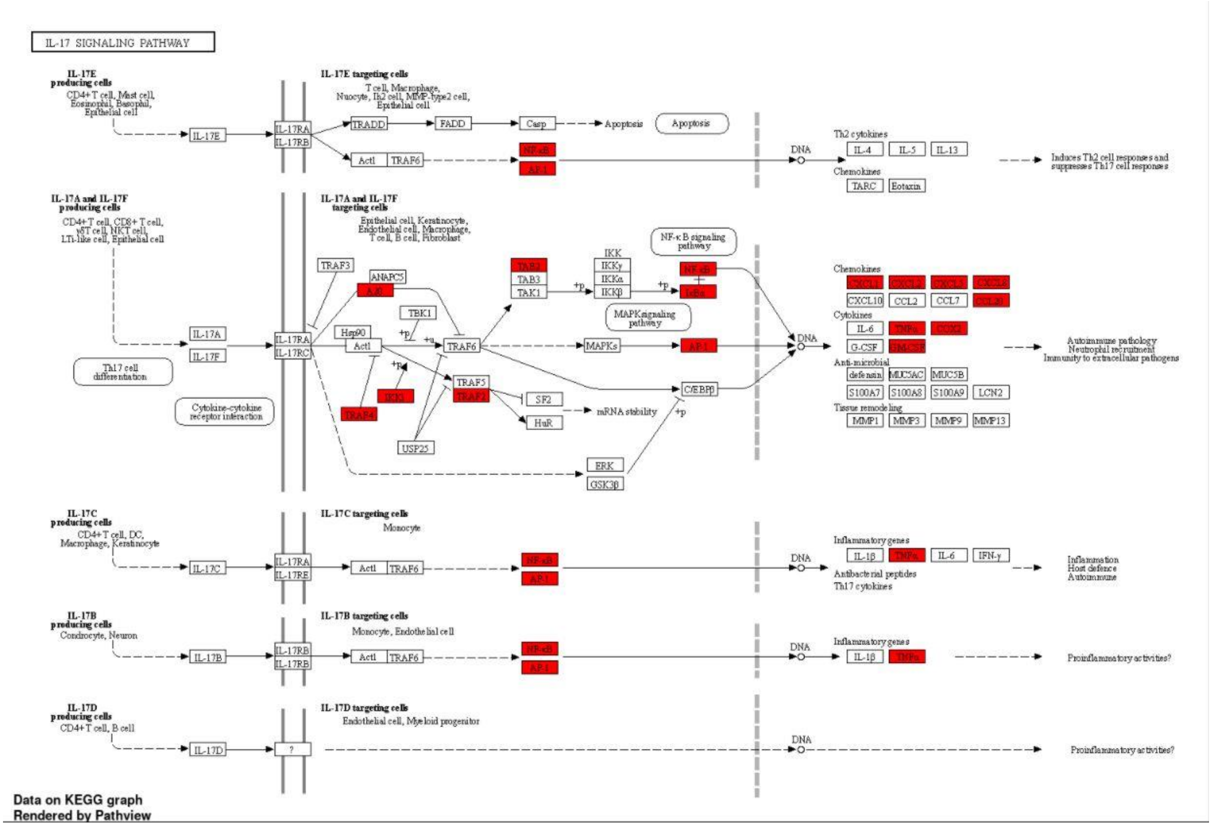
IL-17 SIGNALING PATHWAY
IL-17E producing cells
CD4+ T cell, Mast cell, Eosinophil, Basophil, Epithelial cell
IL-17E targeting cells
T cell, Macrophage, Nuocyte, Th2 cell, MMP-type2 cell, Epithelial cell
IL-17A and IL-17F producing cells
CD4+ T cell, CD8+ T cell, γδT cell, NKT cell, LTi-like cell, Epithelial cell
IL-17A and IL-17F targeting cells
Epithelial cell, Keratinocyte, Endothelial cell, Macrophage, T cell, B cell, Fibroblast
Th17 cell differentiation
Cytokine-cytokine receptor interaction
NF-κB signaling pathway
MAPK signaling pathway
Apoptosis
mRNA stability
Th2 cytokines
Chemokines
Cytokines
Anti-microbial
Tissue remodeling
Induces Th2 cell responses and suppresses Th17 cell responses
Autoimmune pathology
Neutrophil recruitment
Immunity to extracellular pathogens
IL-17C producing cells
CD4+ T cell, DC, Macrophage, Keratinocyte
IL-17C targeting cells
Monocyte
Inflammatory genes
Antibacterial peptides
Th17 cytokines
Inflammation
Host defence
Autoimmune
IL-17B producing cells
Condrocyte, Neuron
IL-17B targeting cells
Monocyte, Endothelial cell
Proinflammatory activities?
IL-17D producing cells
CD4+ T cell, B cell
IL-17D targeting cells
Endothelial cell, Myeloid progenitor
Data on KEGG graph
Rendered by Pathview

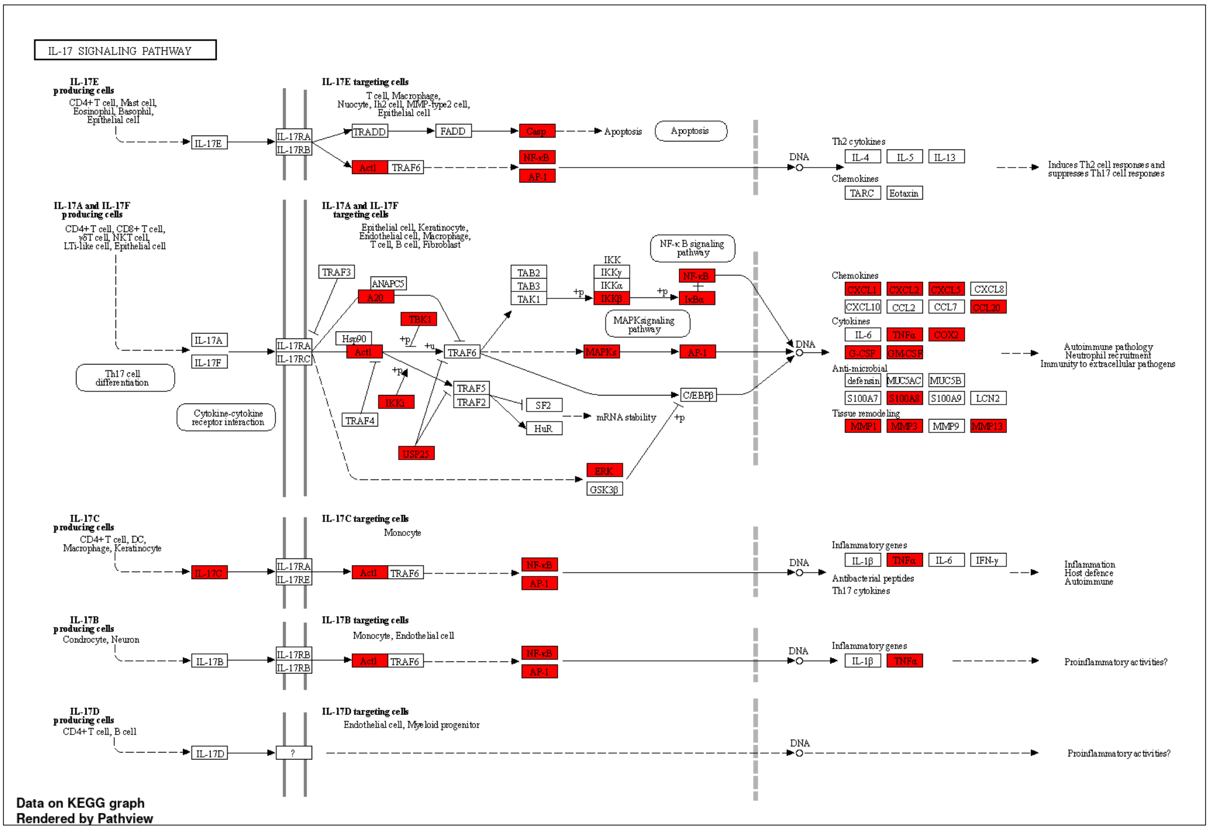
IL-17 SIGNALING PATHWAY
IL-17E producing cells
CD4+ T cell, Mast cell, Eosinophil, Basophil, Epithelial cell
IL-17E targeting cells
T cell, Macrophage, Nuocyte, Th2 cell, MMP-type2 cell, Epithelial cell
IL-17A and IL-17F producing cells
CD4+ T cell, CD8+ T cell, γδT cell, NKT cell, LTi-like cell, Epithelial cell
IL-17A and IL-17F targeting cells
Epithelial cell, Keratinocyte, Endothelial cell, Macrophage, T cell, B cell, Fibroblast
Th17 cell differentiation
Cytokine-cytokine receptor interaction
NF-κB signaling pathway
MAPK signaling pathway
Apoptosis
mRNA stability
Th2 cytokines
Chemokines
Cytokines
Anti-microbial
Tissue remodeling
Induces Th2 cell responses and suppresses Th17 cell responses
Autoimmune pathology
Neutrophil recruitment
Immunity to extracellular pathogens
IL-17C producing cells
CD4+ T cell, DC, Macrophage, Keratinocyte
IL-17C targeting cells
Monocyte
Inflammatory genes
Antibacterial peptides
Th17 cytokines
Inflammation
Host defence
Autoimmune
IL-17B producing cells
Condrocyte, Neuron
IL-17B targeting cells
Monocyte, Endothelial cell
Proinflammatory activities?
IL-17D producing cells
CD4+ T cell, B cell
IL-17D targeting cells
Endothelial cell, Myeloid progenitor
Data on KEGG graph
Rendered by Pathview
