# Supplementary material for: Transcriptomic analysis of intestinal organoids, derived from pigs divergent in feed efficiency, and their response to Escherichia coli
Source: BMC Genomics. 2024 Feb 13;25:173. doi: 10.1186/s12864-024-10064-0 (PMC10863143; doi:10.1186/s12864-024-10064-0)
Supplement: Supplementary file 8 — Additional file 8. DEGs (indicated in red) in the TNF signaling KEGG pathway between unchallenged and challenged colon high (top), colon low (middle) and ileum low (lowest) organoids. Permission for the use of these figures was obtained from KEGG. [file 12864_2024_10064_MOESM8_ESM.pdf]

Additional file 8: DEGs (indicated in red) in the TNF signaling KEGG pathway between unchallenged and challenged colon high (top), colon low (middle) and ileum low (lowest) organoids. Permission for the use of these figures was obtained from KEGG.

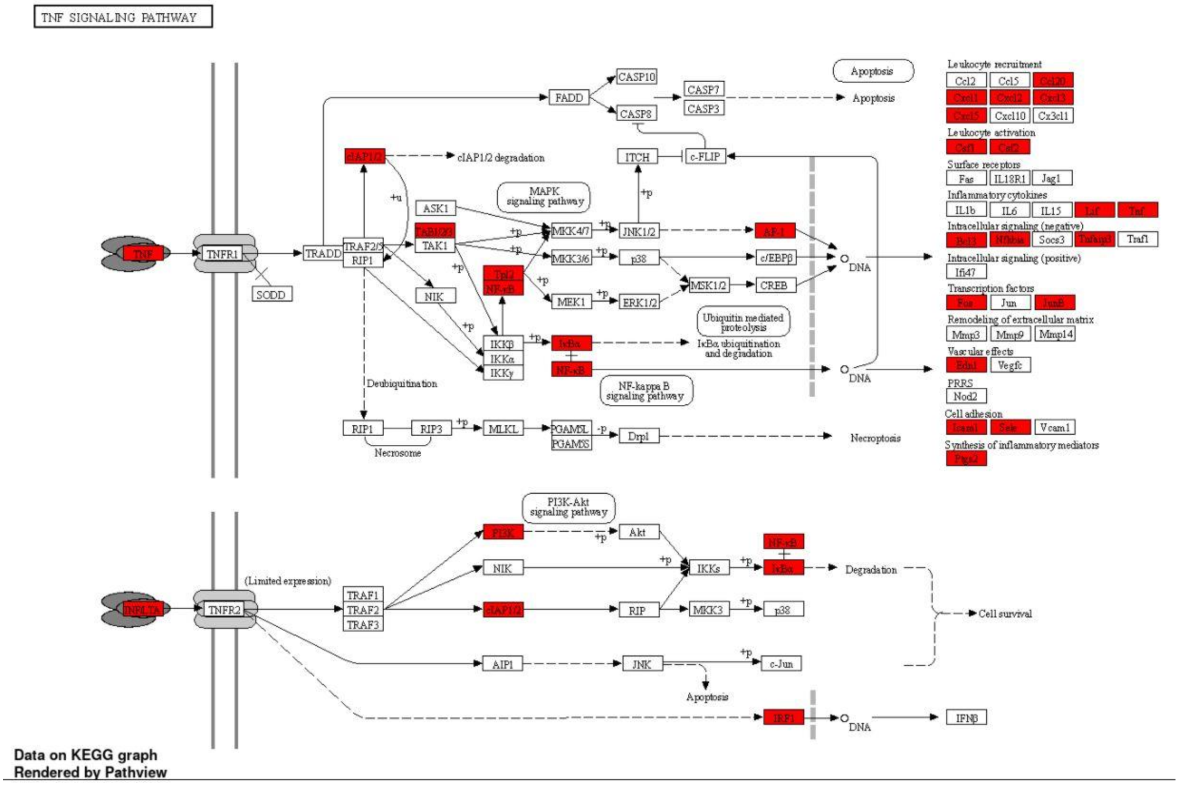

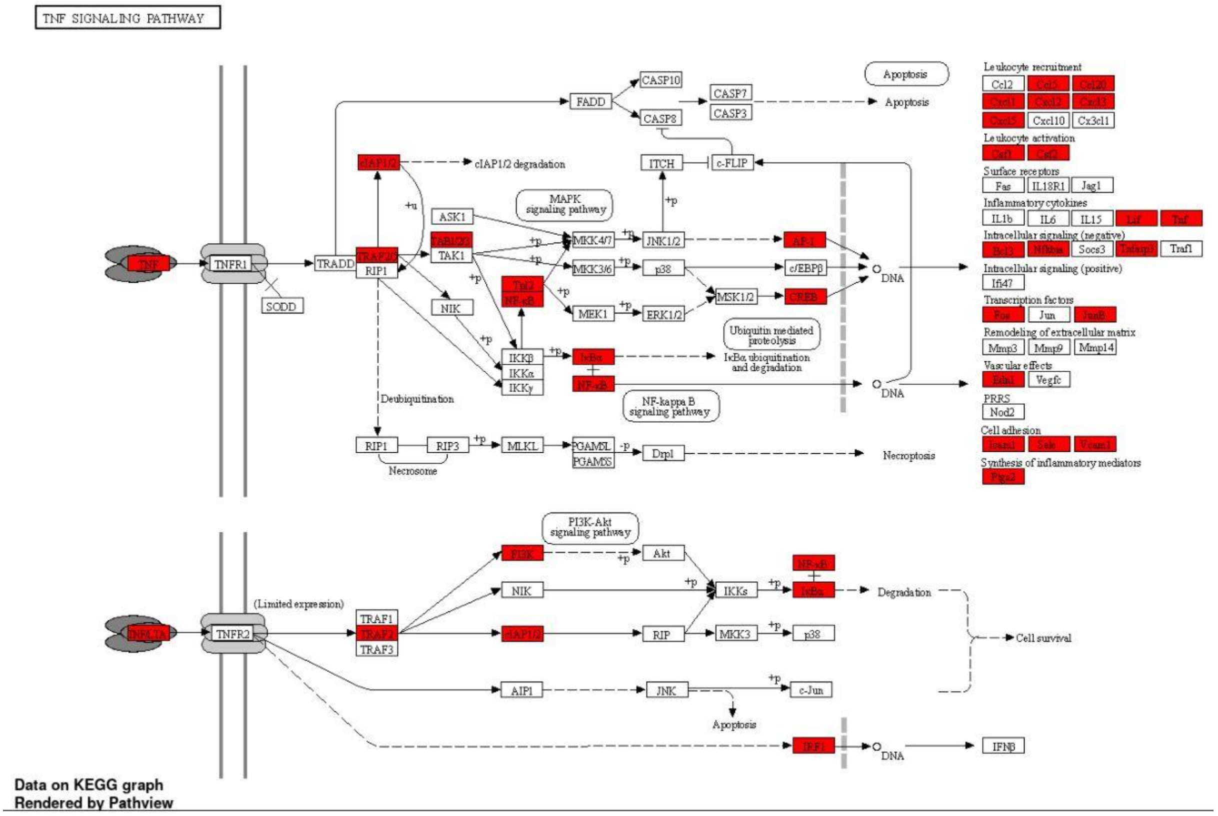
TNF SIGNALING PATHWAY
TNF
TNFR1
TRADD
TRAF2/5
RIP1
SODD
FADD
CASP10
CASP8
CASP7
CASP3
Apoptosis
cIAP1/2
cIAP1/2 degradation
ITCH
c-FLIP
MAPK signaling pathway
ASK1
TAB1/2/3
TAK1
MKK4/7
MKK3/6
JNK1/2
p38
AP-1
c/EBPβ
MSK1/2
CREB
NIK
Tpl2
NF-κB
MEK1
ERK1/2
IKKβ
IKKα
IKKγ
IκBα
Ubiquitin mediated proteolysis
IκBα ubiquitination and degradation
NF-kappa B signaling pathway
DNA
Deubiquitination
RIP3
MLKL
PGAM5L
PGAM5S
Drp1
Necroptosis
Necrosome
Leukocyte recruitment
Leukocyte activation
Surface receptors
Inflammatory cytokines
Intracellular signaling (negative)
Intracellular signaling (positive)
Transcription factors
Remodeling of extracellular matrix
Vascular effects
PRRS
Cell adhesion
Synthesis of inflammatory mediators
PI3K-Akt signaling pathway
PI3K
Akt
IKKs
Degradation
Cell survival
LTA
TNFR2
(Limited expression)
TRAF1
TRAF2
TRAF3
RIP
MKK3
AIP1
JNK
c-Jun
IRF1
IFNβ
Data on KEGG graph
Rendered by Pathview

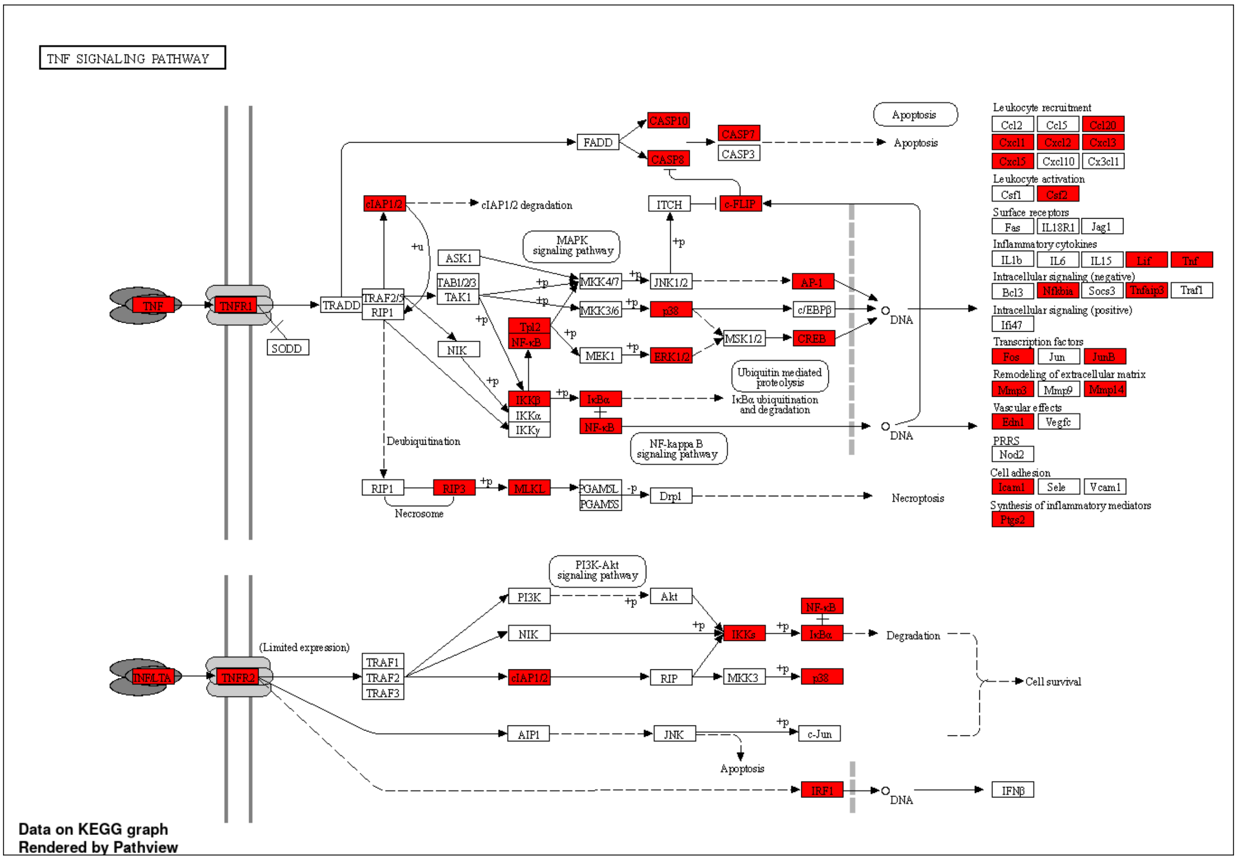
TNF SIGNALING PATHWAY
TNF
TNFR1
TRADD
TRAF2/5
RIP1
SODD
FADD
CASP10
CASP8
CASP7
CASP3
Apoptosis
cIAP1/2
cIAP1/2 degradation
ITCH
c-FLIP
MAPK signaling pathway
ASK1
TAB1/2/3
TAK1
MKK4/7
MKK3/6
JNK1/2
p38
AP-1
c/EBPβ
MSK1/2
CREB
NIK
Tpl2
NF-κB
MEK1
ERK1/2
IKKβ
IKKα
IKKγ
IκBα
Ubiquitin mediated proteolysis
IκBα ubiquitination and degradation
NF-kappa B signaling pathway
DNA
Deubiquitination
RIP3
MLKL
PGAM5L
PGAM5S
Drp1
Necroptosis
Necrosome
Leukocyte recruitment
Leukocyte activation
Surface receptors
Inflammatory cytokines
Intracellular signaling (negative)
Intracellular signaling (positive)
Transcription factors
Remodeling of extracellular matrix
Vascular effects
PRRS
Cell adhesion
Synthesis of inflammatory mediators
PI3K-Akt signaling pathway
PI3K
Akt
IKKs
Degradation
Cell survival
TNFR2
(Limited expression)
TRAF1
TRAF2
TRAF3
RIP
MKK3
AIP1
JNK
c-Jun
IRF1
IFNβ
Data on KEGG graph
Rendered by Pathview
