# Supplementary figures and images for: Transcriptomic analysis of intestinal organoids, derived from pigs divergent in feed efficiency, and their response to Escherichia coli
Source: BMC Genomics. 2024 Feb 13;25:173. doi: 10.1186/s12864-024-10064-0 (PMC10863143; doi:10.1186/s12864-024-10064-0)

- 4

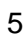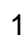

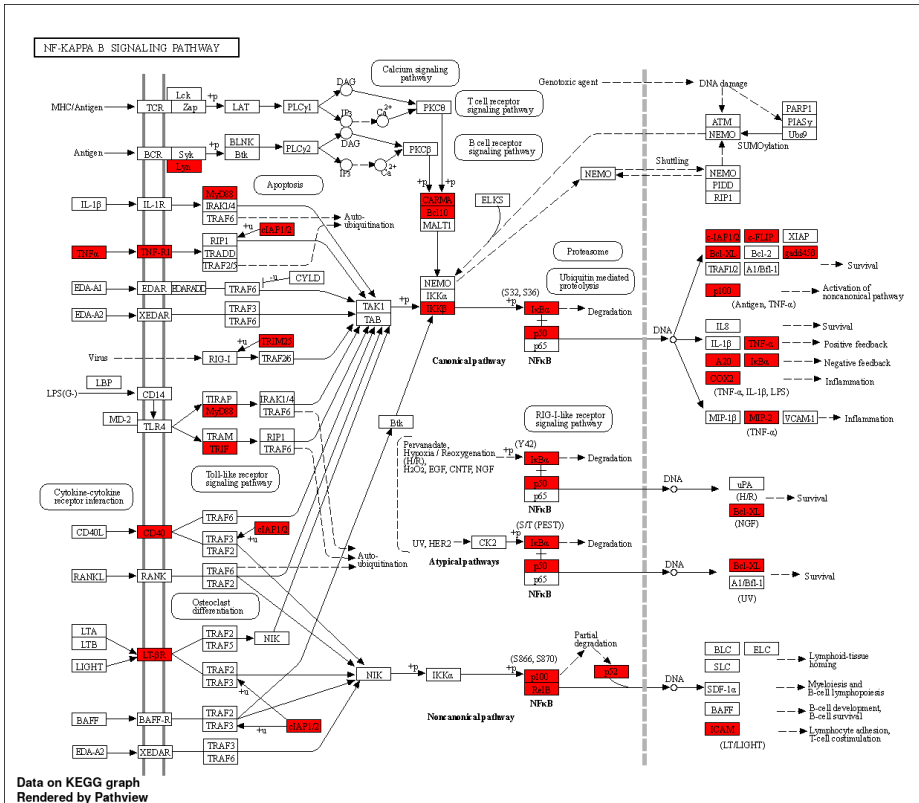

6

Supplement: Supplementary file 9 — Additional file 9. DEGs (indicated in red) in the NF-kappa B signaling KEEG pathway between unchallenged and challenged colon high (top), colon low (middle) and ileum low (lowest) organoids. Permission for the use of these figures was obtained from KEGG. [file 12864_2024_10064_MOESM9_ESM.pdf]

- 4

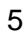

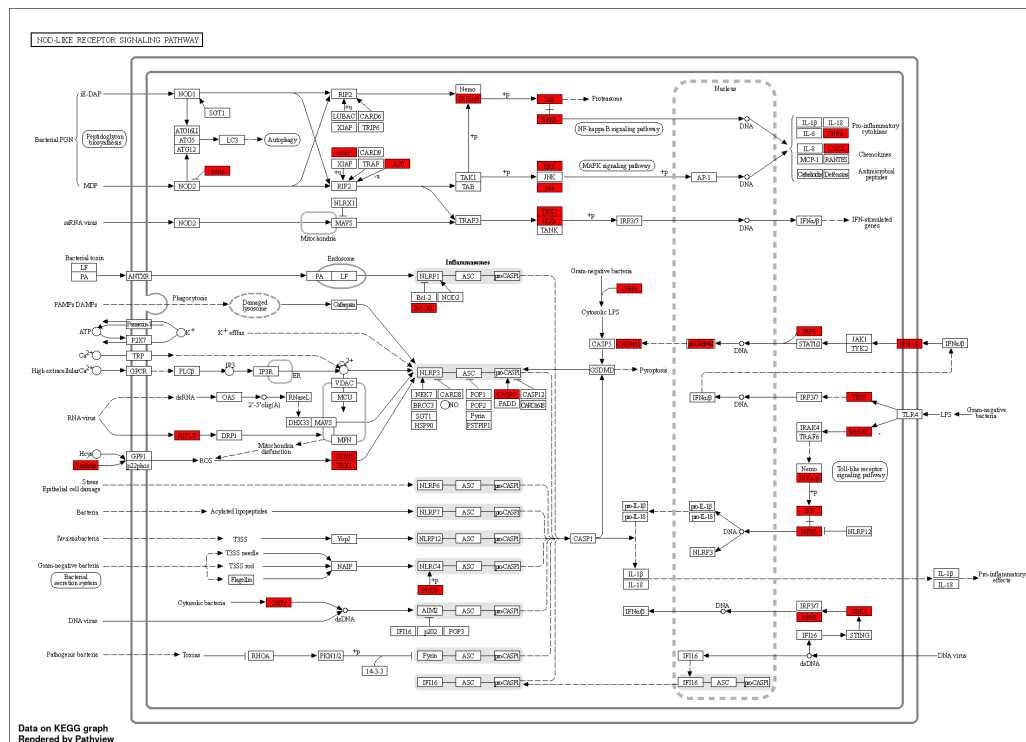

Supplement: Supplementary file 10 — Additional file 10. DEGs (indicated in red) in the NOD signaling KEGG pathway between unchallenged and challenged colon high (top), colon low (middle) and ileum low (lowest) organoids. Permission for the use of these figures was obtained from KEGG. [file 12864_2024_10064_MOESM10_ESM.pdf]
